# The comparison of molecular and morphology-based phylogenies of trichaline net-winged beetles (Coleoptera: Lycidae: Metriorrhynchini) with description of a new subgenus

**DOI:** 10.7717/peerj.3963

**Published:** 2017-10-23

**Authors:** Matej Bocek, Ladislav Bocak

**Affiliations:** Department of Zoology, Faculty of Science, Palacky University, Olomouc, Czech Republic

**Keywords:** Molecular phylogeny, Morphology, Oriental region, Australian region, Phylogeny, New taxa, New synonym

## Abstract

Separate morphological and molecular phylogenetic analyses are presented and the classification of trichaline net-winged beetles is revised. The clade, earlier given a subfamily, tribe or subtribe rank, is a terminal lineage in Metriorrhynchina and contains *Diatrichalus* Kleine, 1926, *Eniclases* Waterhouse, 1879, *Flabellotrichalus* Pic, 1921, *Lobatang* Bocak, 1998, *Microtrichalus* Pic, 1921, *Schizotrichalus* Kleine, 1926, and *Trichalus* Waterhouse, 1877. *Maibrius* subgen. nov. is proposed in *Flabellotrichalus* with the type-species *Flabellotrichalus* (*Maibrius*) *horaki* sp. nov. Unlike previous studies, *Lobatang* is included in the trichaline clade. Further, *Spinotrichalus* Kazantsev, 2010, stat. nov. is down-ranked to the subgenus in *Lobatang* Bocak, 1998 and a new combination, *Lobatang* (*Spinotrichalus*) *telnovi* (Kazantsev, 2010) comb. nov., is proposed. The morphology does not provide a sufficient support for robust phylogeny due to the intrageneric variability of most phenotypic traits and the limited number of characters supporting deep relationships. Most morphological generic diagnoses must be based on the shape of male genitalia. Other characters, such as the shapes of pronotum and antennae are commonly variable within genera. The fronto-lateral pronotal ridges of *Eniclases* + *Schizotrichalus* resemble the ancestral condition in Metriorrhynchini and they re-evolved in the terminal clade and do not indicate the early split of *Eniclases* + *Schizotrichalus* from other trichaline genera. The evolution of morphological traits and the conflict in the morphological and molecular phylogenetic signal are discussed in details. We suggest that the general appearance is affected by the evolution of mimetic complexes, the patterns of elytral costae by their strengthening function, and the presence of flabellate antennae by their role in sexual communication. Then, similar phenotypic traits evolve in unrelated lineages. The results demonstrate that phylogenetic classification must be based on all available information because neither morphological traits nor DNA data robustly support all recovered relationships.

## Introduction

Based on morphological uniqueness, the trichaline genera were given various family group ranks from the subfamily to subtribe (Kleine, 1928; Kleine, 1933a; Bocak & Bocakova, 1990; Bocak, 2002). The molecular analyses recovered these genera as a terminal lineage in the subtribe Metriorrhynchina and to remedy this, they lost their formal rank (Sklenarova, Kubecek & Bocak, 2014). Although most of them are easily recognizable by a single lanceolate pronotal areola and a shortened elytral costa 1 (Kleine, 1928), the limits of the trichaline clade were questioned once the morphology was studied in detail (Bocak, 1998a; Bocak, 2002). Based on the morphological cladistic analysis, *Leptotrichalus* Kleine, 1925, and *Lobatang* Bocak, 1998a were excluded and *Enylus* Waterhouse, 1879, which is now a part of *Synchonnus* Waterhouse, 1879 (Kusy, Sklenarova & Bocak, in press), was recovered as a member of Trichalini (Bocak, 2002). Sklenarova, Kubecek & Bocak (2014) revised the classification of Metriorrhynchini, but only *Trichalus* Waterhouse, 1877, and *Microtrichalus* Pic, 1921b were included in their analyses.

The trichaline clade contains approximately 230 formally described species and these represent ∼20% of Metriorrhynchina diversity. There are high numbers of undescribed taxa in the various regions, as shown by recent studies (Bocak & Bocakova, 1991; Kazantsev, 2010; Bocek & Bocak, 2016; Bocek, 2017; Kusy, 2017). The trichaline species are currently placed in seven genera: *Diatrichalus* Kleine, 1926, *Eniclases* Waterhouse, 1879, *Flabellotrichalus* Pic, 1921b, *Microtrichalus*, *Schizotrichalus* Kleine, 1926, *Trichalus*, and, as shown below, *Lobatang*. The high variability of traditionally used phenotypic characters, especially variable general appearance, modifications of elytral costae and diverse morphology of male antennae, led to the description of a large number of genera in this clade (Kleine, 1926; Pic, 1921b, 1923, 1926, 1930).

The center of trichaline diversity is located in the wet areas of the Australian region: the eastern coast of Australia (40 spp.), New Guinea (131 spp.), and Wallacea (31 spp.). Only a low number of species reach the Oriental region, mainly the Philippines (nine spp.), and the Greater Sundas (22 spp.). Several Indo-Burman species reach as far as the south of the Palearctic region (Kleine, 1933a; Bocak, 1998b, 1999a). The first Australian representatives were already described from specimens brought to Europe in the time of discovery expeditions to the Southern Seas (Fabricius, 1775; Boisduval, 1835). Further species were described in the 19th century, many in other metriorrhynchine genera (Erichson, 1842; Blanchard, 1856; Kirsch, 1875; Macleay, 1886, 1887; Fairmaire, 1877; Waterhouse, 1877, 1878, 1879; Bourgeois, 1900). A. M. Lea, R. Kleine, and M. Pic described over 150 species mainly in 1920s and 1930s (Lea, 1909; Kleine, 1925, 1926, 1930, 1936, 1939; Pic, 1921a, 1921b, 1923, 1926, 1930). *Diatrichalus* and *Microtrichalus* were partly revised in a series of geographically restricted revisions (Bocak & Bocakova, 1991; Bocak, 1998b, 1999a, 2000, 2001). Later, only a single genus, *Spinotrichalus*, and four trichaline species, were described by Kazantsev (2010).

A growing amount of DNA data is currently available for the molecular phylogeny reconstruction of trichaline genera (Sklenarova, Kubecek & Bocak, 2014; Bocek & Bocak, 2016). The aim of this study is to use morphology and molecular phylogeny for the delimitation of genera and build a hypothesis on their relationships. The generic classification should reflect the best supported phylogenetic hypothesis, include only the monophyletic taxa, and be stable. Simultaneously, the genera should also be reliably identified in practice by the evaluation of phenotypic traits (Vences et al., 2013), ideally in the field, or by using simple laboratory equipment. Therefore, we discuss in detail the phenotypic diversification of trichaline genera and the usefulness of various morphological characters for both, phylogenetic inference and diagnostic purposes.

## Materials and Methods

### Sampling, laboratory procedures, and sequence handling

The trichaline net-winged beetles included in current molecular analyses are listed in Table 1. Most terminals in the dataset are identified to the genus level only due to the ambiguous alpha-taxonomy and a high proportion of undescribed species in the dataset. Total DNA was isolated from ethanol-preserved individuals using Wizard SV96 DNA purification system (Promega Inc., Madison, WI, USA). All samples were sequenced for three mtDNA markers: *rrnL* + tRNA-Leu + *nad1* (∼800 bp), *cox1* + tRNA-Leu + *cox2* (∼1,100 bp), and *nad5* + tRNAs (∼1,210 bp; the fragments are further referred as *rrnL*, *cox1*, and *nad5*) using primers reported by Bocak et al. (2008) and Sklenarova, Chesters & Bocak (2013). The chromatograms were edited using the Sequencher 4.9 software package (Gene Codes Corp., Ann Arbor, MI, USA). The newly reported sequences were submitted to GenBank under Accession Numbers MF288149–MF288557 and MF997538–MF997543 (Table 1). Altogether 21 taxa were chosen from previous publication as outgroups. These represent all known Metriorrhynchina major lineages as identified by Bocak et al. (2008), Sklenarova, Chesters & Bocak (2013), and Sklenarova, Kubecek & Bocak (2014). We avoided inclusion of all known ∼150 Metriorrhynchini species available in public databases, as we did not intend to repeat the thorough analysis of the Metriorrhynchini published earlier. Additionally, the high number of distantly related taxa may affect the relationships within ingroup and affect its internal topology as demonstrated by Bocak et al. (2014).

**Table 1:** List of taxa.

Genus, species	Geographic origin	Voucher	Mitochondrial DNA fragments		
		UPOL	*rrnL*	*cox1*	*nad5*
**Outgroup**					
*Cautires* sp.	Malaysia, Pahang, Tanah Rata	000088	KC538654	KC538268	KC538460
*Cautires* sp.	Sumatra, Jambi, Gn Tujuh	000206	KC538676	KC538292	KC538483
*Cautires* sp.	Borneo, Tenggah, Muara Teweh	000262	KC538685	KC538300	KC538491
*Cautires* sp	Borneo, Selatan, Loksado	000342	KC538695	KC538310	KC538501
*Porrostoma* sp.	Australia, Queensland, Lamington	A00035	KC538725	KC538341	KC538532
*Porrostoma* sp.	Australia, Queensland, Lamington	A00042	–	KC538348	KC538539
*Leptotrichalus* sp.	Java, Timor, Sodong	A00451	MF288196	MF288334	MF288457
*Metriorrhynchus* sp.	Sulawesi, Tenggah, Sabbang	000011	KC538629	DQ144660	DQ144686
*M. lineatus*	Sumatra, South, Danau Ranau	000009	KC538628	DQ904297	DQ904259
*M. lobatus*	Sulawesi, Tenggah, Pendolo	000017	KC538630	DQ144662	DQ144688
*M. sericans*	Laos, Houa Phan, Phou Pan	A00381	MF288191	MF288329	MF288452
*Metriorrhynchus* sp.	Australia, Queensland, Lamington	A00043	KC538732	KC538349	KC538540
*Metriorrhynchus* sp.	Malaysia, Johor, Kota Tinggi	A00049	KC538736	KC538354	KC538545
*Metriorrhynchus* sp.	Australia, Queensland, Bunya Mts.	A00311	MF288174	MF288312	MF288437
*Metriorrhynchus* sp.	Australia, Queensland, Lamington	A00348	MF288183	MF288320	MF288445
*Metriorrhynchus* sp.	New Guinea, Biak, Korim	A00422	MF288192	MF288330	MF288453
*Metriorrhynchus* sp.	New Guinea, Papau, Yiwika	BM0104	MF288227	MF288351	MF288487
Metriorrhynchina sp.	New Guinea, West Papua, Maibri	BM0083	MF997538	MF997540	MF997542
Metriorrhynchina sp.	New Guinea, Papua, Yiwika	BM0109	MF997539	MF997541	MF997543
*Synchonnus* sp.	Australia, Queensland, Lamington	A00039	KC538729	KC538345	KC538536
*Wakarumbia* sp.	Sulawesi, Mamasa	MD0155	KC538809	KC538432	KC538624
**Ingroup**					
*Diatrichalus* sp. A	Sulawesi, Selatan, Mamasa	JB0774	–	MF288416	–
*Diatrichalus* sp. B	Malaysia, Kelantan, Kp. Raja	JB0829	–	MF288417	–
*D. xylobanoides*	New Guinea, Crater Mt., Haia	A00118	–	MF288291	MF288419
*D. dilatatus*	New Guinea, Goroka, Gahavisuka	A00133	MF288151	–	MF288544
*D. mancus*	Australia, Queensland, Pascoe River	A00298	MF288172	MF288311	MF288436
*D. manokwarensis*	New Guinea, West Papua, Maibri	BM0079	MF288216	MF288343	MF288477
*D. mindikensis*	New Guinea, Morobe, Mindik	A00184	MF288160	–	MF288427
*D. robustus*	New Guinea, Papua, Elelim	BM0190	MF288288	MF288412	MF288555
*D. robustus*	New Guinea, Papau, Elelim	BM0191	MF288289	MF288413	MF288556
*D. sinuaticollis*	New Guinea, Papua, Bokondini	BM0114	MF288233	MF288357	MF288550
*Diatrichalus* sp. C	New Guinea, Papua, Yiwika	BM0113	MF288232	MF288356	MF288492
*Diatrichalus* sp. D	New Guinea, Papua, Tikapura	BM0127	MF288245	MF288369	MF288504
*Diatrichalus* sp. E	New Guinea, Papua, Elelim	BM0159	MF288267	MF288391	MF288526
*Diatrichalus* sp. F	New Guinea, Papua, Elelim	BM0192	MF288290	MF288414	MF288557
*Diatrichalus* sp. G	Australia, Queensland, Chilverton	A00208	MF288163	MF288302	MF288546
*Diatrichalus* sp. G	Australia, Queensland, Chilverton	A00237	MF288167	MF288306	MF288547
*Diatrichalus* sp. G	Australia, Queensland, Garradunga	A00308	MF288173	–	–
*Diatrichalus* sp. G	Australia, Queensland, Garradunga	A00337	MF288181	–	MF288548
*Diatrichalus* sp. H	New Guinea, Papua, Tikapura	BM0189	MF288287	MF288411	MF288554
*Diatrichalus* sp. I	New Guinea, Goroka, Gahavisuka	A00131	MF288150	–	–
*Diatrichalus* sp. I	New Guinea, Goroka, Gahavisuka	A00156	MF288154	MF288295	MF288545
*Diatrichalus* sp. J	New Guinea, Papua, Tikapura	BM0188	MF288286	MF288410	MF288553
*Diatrichalus* sp. K	New Guinea, West Papua, Wasior	JB0772	–	MF288415	–
*D. tenimberensis*	Australia, Queensland, Claudie River	A00366	MF288190	MF288328	MF288549
*Eniclases apertus*	New Guinea, Papua, Sentani	BM0018	MF288201	KT265155	MF288462
*E. bicolor*	New Guinea, Papua, Elelim	BM0045	MF288204	KT265166	MF288465
*E. bokondinensis*	New Guinea, Papua, Bokondini	BM0094	MF288222	KT265153	MF288482
*E. brancuccii*	New Guinea, Papua, Sentani	BM0005	MF288199	KT265118	MF288460
*E. divaricatus*	New Guinea, Papua, Sentani	BM0001	MF288197	KT265092	MF288458
*E. divaricatus*	New Guinea, Papua, Elelim	BM0057	MF288207	KT265098	MF288468
*E. elelimensis*	New Guinea, Papua, Elelim	BM0051	MF288206	KT265149	MF288467
*E. infuscatus*	New Guinea, Papua, Elelim	BM0050	MF288205	KT265169	MF288466
*E. niger*	New Guinea, Papua, Bokondini	BM0033	MF288202	KT265111	MF288463
*E. pseudoluteolus*	New Guinea, West Papua, Maibri	BM0084	MF288219	KT265171	MF288480
*E. similis*	New Guinea, Papua, Sentani	BM0003	MF288198	KT265099	MF288459
*Eniclases* sp. A	New Guinea, Papua, Bokondini	BM0093	MF288221	KT265163	MF288481
*E. tikapurensis*	New Guinea, Papua, Yiwika	BM0039	MF288203	KT265157	MF288464
*E. variabilis*	New Guinea, Papua, Sentani	BM0008	MF288200	KT265122	MF288461
*Flabellotrichalus* sp. A	New Guinea, Crater Mt., Haia	A00170	MF288157	MF288298	MF288425
*Flabellotrichalus* sp. B	New Guinea, Pindiu, Mongi	A00180	MF288159	MF288300	MF288426
*Flabellotrichalus* sp. C	New Guinea, Papua, Yiwika	BM0103	MF288226	MF288350	MF288486
*Flabellotrichalus* sp. C	New Guinea, Papua, Yiwika	BM0110	MF288230	MF288354	MF288490
*Flabellotrichalus* sp. C	New Guinea, Papua, Yiwika	BM0111	MF288231	MF288355	MF288491
*Flabellotrichalus* sp. D	New Guinea, Pt. Moresby, Kailaki	A00149	MF288153	MF288294	MF288422
*Flabellotrichalus* sp. D	New Guinea, Papua, Elelim	BM0148	MF288257	MF288381	MF288516
*Flabellotrichalus* sp. D	New Guinea, Papua, Elelim	BM0149	MF288258	MF288382	MF288517
*Flabellotrichalus* sp. D	New Guinea, Papua, Elelim	BM0150	MF288259	MF288383	MF288518
*Flabellotrichalus* sp. E	New Guinea, Crater Mt., Haia	A00172	MF288158	MF288299	–
*Flabellotrichalus* sp. F	New Guinea, Crater Mt., Haia	A00125	MF288149	MF288292	MF288420
*Flabellotrichalus* sp. F	New Guinea, Crater Mt., Haia	A00162	MF288155	MF288296	MF288423
*Flabellotrichalus* sp. F	New Guinea, Crater Mt., Haia	A00169	MF288156	MF288297	MF288424
*Flabellotrichalus* sp. G	Australia, Queensland, Chilverton	A00211	MF288165	MF288304	MF288430
*Flabellotrichalus* sp. H	New Guinea, Papua, Yiwika	BM0105	MF288228	MF288352	MF288488
*Flabellotrichalus* sp. I	New Guinea, Papua, Elelim	BM0151	MF288260	MF288384	MF288519
*F.* (*Maibrius*) *horaki*	New Guinea, West Papua, Maibri	BM0082	MF288218	MF288345	MF288479
*Lobatang* sp. A	New Guinea, Papua, Sentani	BM0162	MF288269	MF288393	MF288528
*Lobatang* sp. A	New Guinea, Papua, Sentani	BM0168	MF288274	MF288398	MF288533
*Lobatang* sp. B	Australia, Queensland, Claudie River	A00363	MF288187	MF288325	MF288450
*Lobatang* sp. B	Australia, Queensland, Claudie River	A00365	MF288189	MF288327	–
*Lobatang* sp. C	Moluccas, Buru isl., Remaja Mt.	BM0071	MF288208	MF288335	MF288469
*Lobatang* sp. C	Moluccas, Buru isl., Remaja Mt.	BM0072	MF288209	MF288336	MF288470
*Lobatang* sp. C	Moluccas, Buru isl., Remaja Mt.	BM0073	MF288210	MF288337	MF288471
*Lobatang* sp.	Moluccas, Buru isl., Remaja Mt.	BM0074	MF288211	MF288338	MF288472
*Lobatang* sp. D	New Guinea, West Papua, Maibri	BM0075	MF288212	MF288339	MF288473
*Lobatang* sp. D	New Guinea, West Papua, Maibri	BM0076	MF288213	MF288340	MF288474
*Lobatang* sp. D	New Guinea, Papua, Elelim	BM0145	MF288254	MF288378	MF288513
*Lobatang* sp. D	New Guinea, Papua, Elelim	BM0146	MF288255	MF288379	MF288514
*Lobatang* sp. D	New Guinea, Papua, Sentani	BM0165	MF288271	MF288395	MF288530
*Lobatang* sp. D	New Guinea, Papua, Sentani	BM0166	MF288272	MF288396	MF288531
*Microtrichalus* sp. A	New Guinea, Papua, Sentani	BM0175	MF288277	MF288401	MF288551
*Microtrichalus* sp. A	New Guinea, Papua, Sentani	BM0180	MF288281	MF288405	MF288552
*Microtrichalus* sp. B	New Guinea, Papua, Sentani	BM0178	MF288279	MF288403	MF288537
*Microtrichalus* sp. B	New Guinea, Papua, Sentani	BM0179	MF288280	MF288404	MF288538
*Microtrichalus* sp. C	Australia, Queensland, Claudie River	A00356	–	MF288322	MF288447
*Microtrichalus* sp. C	Australia, Queensland, Claudie River	A00364	MF288188	MF288326	MF288451
*Microtrichalus* sp. D	New Guinea, Papua, Elelim	BM0158	MF288266	MF288390	MF288525
*Microtrichalus* sp. E	New Guinea, Papua, Tikapura	BM0134	MF288247	MF288371	MF288506
*Microtrichalus* sp. F	New Guinea, Papua, Bokondini	BM0117	MF288236	MF288360	MF288495
*Microtrichalus* sp. F	New Guinea, Papua, Tikapura	BM0135	MF288248	MF288372	MF288507
*Microtrichalus* sp. G	New Guinea, Papua, Yiwika	BM0102	MF288225	MF288349	MF288485
*Microtrichalus* sp. G	New Guinea, Papua, Tikapura	BM0126	MF288244	MF288368	MF288503
*Microtrichalus* sp. H	New Guinea, West Papua, Maibri	BM0077	MF288214	MF288341	MF288475
*Microtrichalus* sp. H	New Guinea, West Papua, Maibri	BM0085	MF288220	MF288346	–
*Microtrichalus* sp. I	New Guinea, Papua, Bokondini	BM0122	MF288241	MF288365	MF288500
*Microtrichalus* sp. I	New Guinea, Papua, Bokondini	BM0123	MF288242	MF288366	MF288501
*Microtrichalus* sp. I	New Guinea, Papua, Elelim	BM0152	MF288261	MF288385	MF288520
*Microtrichalus* sp. I	New Guinea, Papua, Elelim	BM0153	MF288262	MF288386	MF288521
*Microtrichalus* sp. J	Australia, Queensland, Chilverton	A00239	MF288168	MF288307	MF288432
*Microtrichalus* sp. J	Australia, Queensland, Chilverton	A00243	MF288169	MF288308	MF288433
*Microtrichalus* sp. K	New Guinea, Papua, Sentani	BM0160	MF288268	MF288392	MF288527
*Microtrichalus* sp. K	New Guinea, Papua, Sentani	BM0164	MF288270	MF288394	MF288529
*Microtrichalus* sp. K	New Guinea, Papua, Sentani	BM0167	MF288273	MF288397	MF288532
*Microtrichalus* sp. K	New Guinea, Papua, Sentani	BM0169	MF288275	MF288399	MF288534
*Microtrichalus* sp. L	New Guinea, Papua, Elelim	BM0147	MF288256	MF288380	MF288515
*Microtrichalus* sp. M	Australia, Queensland, Claudie River	A00353	MF288184	MF288321	MF288446
*Microtrichalus* sp. N	New Guinea, Papua, Bokondini	BM0119	MF288238	MF288362	MF288497
*Microtrichalus* sp. O	New Guinea, Papua, Napua	BM0185	MF288283	MF288407	MF288540
*Microtrichalus* sp. O	New Guinea, Papua, Tikapura	BM0141	MF288253	MF288377	MF288512
*Microtrichalus* sp. P	Australia, Queensland, Mt. Molloy	000375	KC538702	KC538315	KC538506
*Microtrichalus* sp. P	Australia, Queensland, Pascoe River	A00314	MF288176	MF288314	MF288439
*Microtrichalus* sp. P	Australia, Queensland, Pascoe River	A00315	MF288177	MF288315	MF288440
*Microtrichalus* sp. P	Australia, Queensland, Pascoe River	A00316	MF288178	MF288316	MF288441
*Microtrichalus* sp. Q	Australia, Queensland, Chilverton	A00210	MF288164	MF288303	MF288429
*Microtrichalus* sp. R	New Guinea, Papua, Sentani	BM0183	MF288282	MF288406	MF288539
*Microtrichalus* sp. S	New Guinea, Papua, Bokondini	BM0120	MF288239	MF288363	MF288498
*Microtrichalus* sp. T	Australia, Queensland, Chilverton	A00206	MF288162	MF288301	–
*Microtrichalus* sp. T	Australia, Queensland, Chilverton	A00235	MF288166	MF288305	MF288431
*Microtrichalus* sp. T	Australia, Queensland, Duintrea	A00192	MF288161	–	MF288428
*Microtrichalus* sp. U	New Guinea, Papua, Yiwika	BM0108	MF288229	MF288353	MF288489
*Microtrichalus* sp. V	New Guinea, Papua, Bokondini	BM0115	MF288234	MF288358	MF288493
*Microtrichalus* sp. W	New Guinea, Goroka, Gahavisuka	A00139	MF288152	MF288293	MF288421
*Microtrichalus* sp. X	New Guinea, Papua, Yiwika	BM0100	MF288223	MF288347	MF288483
*Microtrichalus* sp. X	New Guinea, Papua, Napua	BM0186	MF288284	MF288408	MF288541
*Microtrichalus* sp. Y	Australia, Queensland, Claudie River	A00270	MF288170	MF288309	MF288434
*Microtrichalus* sp. Y	Australia, Queensland, Claudie River	A00357	MF288185	MF288323	MF288448
*Microtrichalus* sp. Y	Australia, Queensland, Claudie River	A00362	MF288186	MF288324	MF288449
*Microtrichalus* sp. Z	New Guinea, Papua, Bokondini	BM0121	MF288240	MF288364	MF288499
*Microtrichalus* sp. Z	New Guinea, Papua, Bokondini	BM0124	MF288243	MF288367	MF288502
*Microtrichalus* sp. Z	New Guinea, Papua, Sentani	BM0177	MF288278	MF288402	MF288536
*Microtrichalus* sp. AA	Borneo, Sabah, Poring	MK0852	–	MF288418	MF288543
*Microtrichalus* sp. AB	New Guinea, Papua, Bokondini	BM0116	MF288235	MF288359	MF288494
*Microtrichalus* sp. AB	New Guinea, Papua, Bokondini	BM0118	MF288237	MF288361	MF288496
*Microtrichalus* sp. AC	New Guinea, West Papua, Maibri	BM0081	MF288217	MF288344	MF288478
*Microtrichalus* sp. AD	New Guinea, Papua, Elelim	BM0154	MF288263	MF288387	MF288522
*Microtrichalus* sp. AD	New Guinea, Papua, Elelim	BM0156	MF288264	MF288388	MF288523
*Microtrichalus* sp. AD	New Guinea, Papua, Elelim	BM0157	MF288265	MF288389	MF288524
*Trichalus* sp. A	Australia, Queensland, Lamington	A00032	KC538722	KC538339	KC538529
*Trichalus* sp. B	Australia, Queensland, Tinarooo	A00312	MF288175	MF288313	MF288438
*Trichalus* sp. B	Australia, Queensland, Fletcher Creek	A00320	MF288179	MF288317	MF288442
*Trichalus* sp. B	Australia, Queensland, Mt. Garnet	A00342	MF288182	MF288319	MF288444
*Trichalus* sp. C	Australia, Queensland, Garradunga	A00336	MF288180	MF288318	MF288443
*Trichalus* sp. D	Australia, Queensland, Fletcher Creek	A00287	MF288171	MF288310	MF288435
*T. communis*	Malaysia, Kelantan, Gua Musang	A00425	MF288193	MF288331	MF288454
*T. communis*	Malaysia, Kelantan, Gua Musang	A00426	MF288194	MF288332	MF288455
*Trichalus* sp. E	New Guinea, West Papua, Maibri	BM0078	MF288215	MF288342	MF288476
*Trichalus* sp. F	New Guinea, Papua, Sentani	BM0174	MF288276	MF288400	MF288535
*Trichalus* sp. G	New Guinea, Papua, Napua	BM0187	MF288285	MF288409	MF288542
*Trichalus* sp. H	New Guinea, Papua, Tikapura	BM0136	MF288249	MF288373	MF288508
*Trichalus* sp. H	New Guinea, Papua, Tikapura	BM0140	MF288252	MF288376	MF288511
*Trichalus* sp. I	New Guinea, Papua, Tikapura	BM0133	MF288246	MF288370	MF288505
*Trichalus* sp. J	New Guinea, Papua, Tikapura	BM0138	MF288250	MF288374	MF288509
*Trichalus* sp. J	New Guinea, Papua, Yiwika	BM0101	MF288224	MF288348	MF288484
*Trichalus* sp. J	New Guinea, Papua, Tikapura	BM0139	MF288251	MF288375	MF288510

**Note:**

The list of terminals in the molecular phylogenetic analyses, with voucher and GenBank accession numbers.

All voucher specimens, including the type material, are deposited in the voucher collection of the Department of Zoology, Palacky University in Olomouc, Czech Republic (LMBC).

### Phylogenetic analyses of the molecular dataset

Each DNA fragment was separately aligned with MAFFT 7.017 plug-in (Katoh & Standley, 2013) in Geneious R7.1.9 (Biomatters Inc., Newark, NJ, USA) and G-Ins-i algorithm. The alignment of the protein-coding genes *cox1*, *cox2*, *nad1*, and *nad5* were checked by amino acid reading frames and manually corrected, if necessary. The concatenated supermatrix was partitioned using PartitionFinder2 for all fragments and codon positions when appropriate (Lanfear et al., 2014, 2016). The following partitions and models were proposed for the maximum-likelihood (ML) and Bayesian analyses. The RAxML best partitioning scheme: 13 subsets; 1 = 1–617, 2 = 618–684, 1,592–1,651, 3 = 1,912–2,925\3, 685–808\3; 4 = 686–808\3, 1,913–2,925\3, 5 = 687–808\3, 6 = 809–1,591\3, 7 = 810–1,591\3, 8 = 811–1,591\3, 9 = 1,652–1,911\3, 10 = 1,653–1,911\3, 11 = 1,654–1,911\3, 12 = 1,914–2,925\3, 13 = 2,926–3,184. The model GTR+I+G was proposed for subsets 1–9 and 13 and GTR+G for subsets 10–12. The model GTR+I+G was applied for all subsets in the maximum-likelihood analyses as RAxML allows for only a single model of rate heterogeneity in partitioned analyses. I.e., we assigned GTR+I+G as the model providing the most accurate estimation of the DNA evolution (Stamatakis, 2014; Lanfear et al., 2014, 2016). The position cited refers to those in the supermatrix provided as the File S1, i.e., the aligned DNA dataset used for the ML analysis. The BI best partitioning scheme: 14 subsets; 1 = 1–617, 2 = 618–684, 1,592–1,651, 3 = 1,912–2,925\3, 685–808\3, 4 = 686–808\3, 5 = 687–808\3, 6 = 809–1,591\3, 7 = 810–1,591\3, 8 = 811–1,591\3, 9 = 1,652–1,911\3, 10 = 1,653–1,911\3, 11 = 1,654–1,911\3, 12 = 1,913–2,925\3, 13 = 1,914–2,925\3, 14 = 2,926–3,184. The model GTR+I+G was proposed for subsets 1–9, 13–14 and GTR+G for subsets 10–12. The models were applied in the BI analysis as proposed by PartitionFinder2. The position refers to the alignment provided in the File S1 as above.

We used the ML criterion and Bayesian interference (BI) for phylogenetic analyses of the partitioned supermatrix (File S1). The ML searches were conducted in RAxML 8.2.10 (Stamatakis, 2014) on the CIPRES cluster (Miller, Pfeiffer & Schwartz, 2010) with the partitions described above and the GTR+I+G model identified using PartitionFinder2 as described above. Additionally, we analyzed the dataset with the partition by genes and protein coding positions when appropriate and the GTR+I+G model identified by jModelTest 2.1.7 (Darriba et al., 2012). Bootstrap support values were calculated in both analyses from 1,000 pseudoreplicates using the GTR+I+G model proposed by PartitionFinder2 or using the GTRCAT model which enables a time-effective and still sufficiently precise estimation of the bootstrap support in the analysis using partitions by genes (Stamatakis, 2014). The BI analysis was run in MrBayes 3.2.6 (Ronquist et al., 2012) on the CIPRES cluster under the best partitioning scheme suggested by PartitionFinder2 (Lanfear et al., 2014, 2016; see above) for 6 × 10^7^ generations, sampling a single tree every 1,200 generations. The first 5,000 trees were discarded as burn-in after the identification of the stationary phase and the effective sample size in Tracer 1.6 (Rambaut et al., 2014). The same analysis was run with gene partitions and GTR+I+G model as proposed by jModelTest 2.1.7 (Darriba et al., 2012). Posterior probabilities (PP) were calculated from the post-burn-in trees and mapped on the maximum credibility tree. Both trees produced by ML and BI analyses were rooted by *Cautires* Waterhouse, 1879 (the type genus of the sister subtribe Cautirina, see Bocak et al., 2008; Sklenarova, Chesters & Bocak, 2013; Sklenarova, Kubecek & Bocak, 2014). The rooting forces Metriorrhynchina to be a clade, but we do not force trichaline genera to be monophyletic and their monophyly can be rigorously re-tested by the current analysis. All trees were visualized in FigTree 1.4.2 (http://tree.bio.ed.ac.uk/software/figtree) and edited in a graphic software.

### Morphological phylogeny

Adult semaphoronts were used for morphological descriptions. Male and female genitalia were relaxed and cleared in hot 10% KOH, dissected and stained by chlorazol black when needed. All photographs were taken using a camera on an Olympus SZX-16 binocular microscope. The morphological measurements were taken with the ocular scale.

The characters from earlier published morphological datasets (Bocak, 1998a, 2002) and the newly identified characters (Kazantsev, 2010) were compiled in a single dataset of 11 taxa and 28 characters (Table 2; File S2). *Metriorrhynchus* was considered as an outgroup when the tree was rooted. The characters in the trichaline clade were polarized by the outgroup criterion. The autapomorphies of genera are based on inspection of all available taxa classified in the respective genus and they are included in the analysis to map their distribution. These characters do not affect the topology. The following characters were coded for all genera of the trichaline clade and taxa representing non-trichaline Metriorrhynchina:

1. **Shape of external mandibular margin in ventral view:** (0) nearly straight; (1) concave.
2. **Shape of mandibles:** (0) slightly curved or sickle-shaped; (1) apical part curved in right angle.
3. **Shape of mandibular incisor:** (0) inner margin twice broken; (1) inner margin continuously curved.
4. **Shape of apical maxillary palpomere:** (0) securiform; (1) parallel-sided, more or less obliquely cut at apex.
5. **Presence of sensillae at apex of terminal palpomere:** (0) absent; (1) present.
6. **Shape of male antennae:** (0) male antennae filiform to serrate; (1) antennomeres 3–10 flabellate.
7. **Shape of pronotum:** (0) approximately as long as wide; (1) much longer than wide.
8. **Pubescence of pronotum:** (0) whole pronotum with pubescence of the same type and density; (1) apparently denser and longer pubescence at lateral and frontal margins.
9. **Strength of hind margin of metascutellum:** (0) hind margin of metascutellum simple; (1) bent, strengthened.
10. **Shape of hind margin of metascutellum and presence of the metascutellar keel:** (0) hind margin of metascutellum straight, without keel; (1) emarginate, with keel.
11. **Arrangement of pronotal carinae:** (0) seven pronotal areolae; (1) less than seven pronotal areolae.
12. **Number of pronotal areolae:** (0) at least five areolae or at least vestiges of frontal and postero-lateral keels present; (1) only a lanceolate median areola present.
13. **Strengthened pronotal longitudinal carinae:** (0) absent; (1) present.
14. **The number of fully developed elytral primary costae in middle part of elytron:** (0) four primary costae; (1) three primary costae.
15. **Secondary elytral costae:** (0) secondary costae present; (1) absent.
16. **Split tarsal claws:** (0) no; (1) yes.
17. **Shape of apical part of phallus:** (0) wider or as wide as its middle part, only in apical part open, if apical part slender, then well-sclerotized and internal sac widely exposed; (1) apical part of phallus slender, with cup-shaped apex, only dorsal part sclerotized.
18. **Phallus short, robust, sometimes with a ventral process**: (0) no; (1) yes.
19. **Sickle-shaped thorns at base of internal sac:** (0) absent; (1) present.
20. **Single keel in dorsal part of phallus:** (0) absent; (1) present.
21. **Internal sac:** (0) membranous or with sclerotized sclerites in apical part; (1) rod-shaped at least in the basal part.
22. **Internal sac with y-shaped base:** (0) no; (1) yes.
23. **Shape of valvifers:** (0) valvifers long, slender; (1) valvifers short, fused with coxites.
24. **Attachment of lateral vaginal glands:** (0) laterally; (1) dorsally.
25. **Lateral pockets on vagina:** (0) absent; (1) present.
26. **Unpaired slim vaginal gland:** (0) absent; (1) present.
27. **Length of spermatheca:** (0) relatively short, lemon-like; (1) long, slender.
28. **Structure of the basal part of the spermathecal duct:** (0) slim; (1) robust.

**Table 2:** Morphological dataset.

Characters	0000000001111111111222222222
Taxa	1234567890123456789012345678
*Metriorrhynchus*	00000-0000000000000000000000
*Kassemia*	0010010000000110000000000010
*Synchonnus*	0110000000110000001000000010
*Diatrichalus*	01101000011101-0010000000011
*Leptotrichalus*	0001001000110100000000100000
*Lobatang*	00000000001101010000101000-0
*Schizotrichalus*	01010000101001-0100100010000
*Eniclases*	11010-0010101100100100010000
*Flabellotrichalus*	1101010100110100100001010000
*Trichalus*	1100000001110100101-00010000
*Microtrichalus*	1101000001110100101000011100

**Note:**

The description of character states is provided in the text.

The maximum parsimony (MP) analysis was performed using PAUP* 4.0 (Swofford, 2002). Heuristic searches were conducted with 1,000 repetitions and random stepwise additions; all characters were unordered and equally weighted and polymorphic characters were treated as “missing” data. The level of confidence in each node of the MP trees was assessed using bootstrapping based on 1,000 pseudoreplicates, each analysis with 100 random additions. Further, we estimated morphology-based phylogenetic relationships using Bayesian inference as implemented in BEAST 2 (Bouckaert et al., 2013). The analysis was conducted using Lewis MK substitution model, a lognormal relaxed clock model, and a birth–death tree prior. The number of generation was set to 10^7^ and sampling frequency every 1,000 generation. We used Tracer 1.6 (Rambaut et al., 2014) to confirm convergence, and based on this, we discarded the first 25% of generations as burn-in. We used the program TreeAnnotator 2.4.5 (Bouckaert et al., 2013) to produce maximum clade credibility tree with PP.

The electronic version of this article in portable document format (PDF) will represent a published work according to the International Commission on Zoological Nomenclature (ICZN), and hence the new names contained in the electronic version are effectively published under that Code from the electronic edition alone. This published work and the nomenclatural acts it contains have been registered in ZooBank, the online registration system for the ICZN. The ZooBank LSIDs (Life Science Identifiers) can be resolved and the associated information viewed through any standard web browser by appending the LSID to the prefix http://zoobank.org/. The LSID for this publication is: urn:lsid:zoobank.org:pub:BCDB57BC-DF3E-42A8-AB6D-2DCAB44799F3. The online version of this work is archived and available from the following digital repositories: PeerJ, PubMed Central and CLOCKSS.

## Results

### Molecular analysis

**Figure 1:**
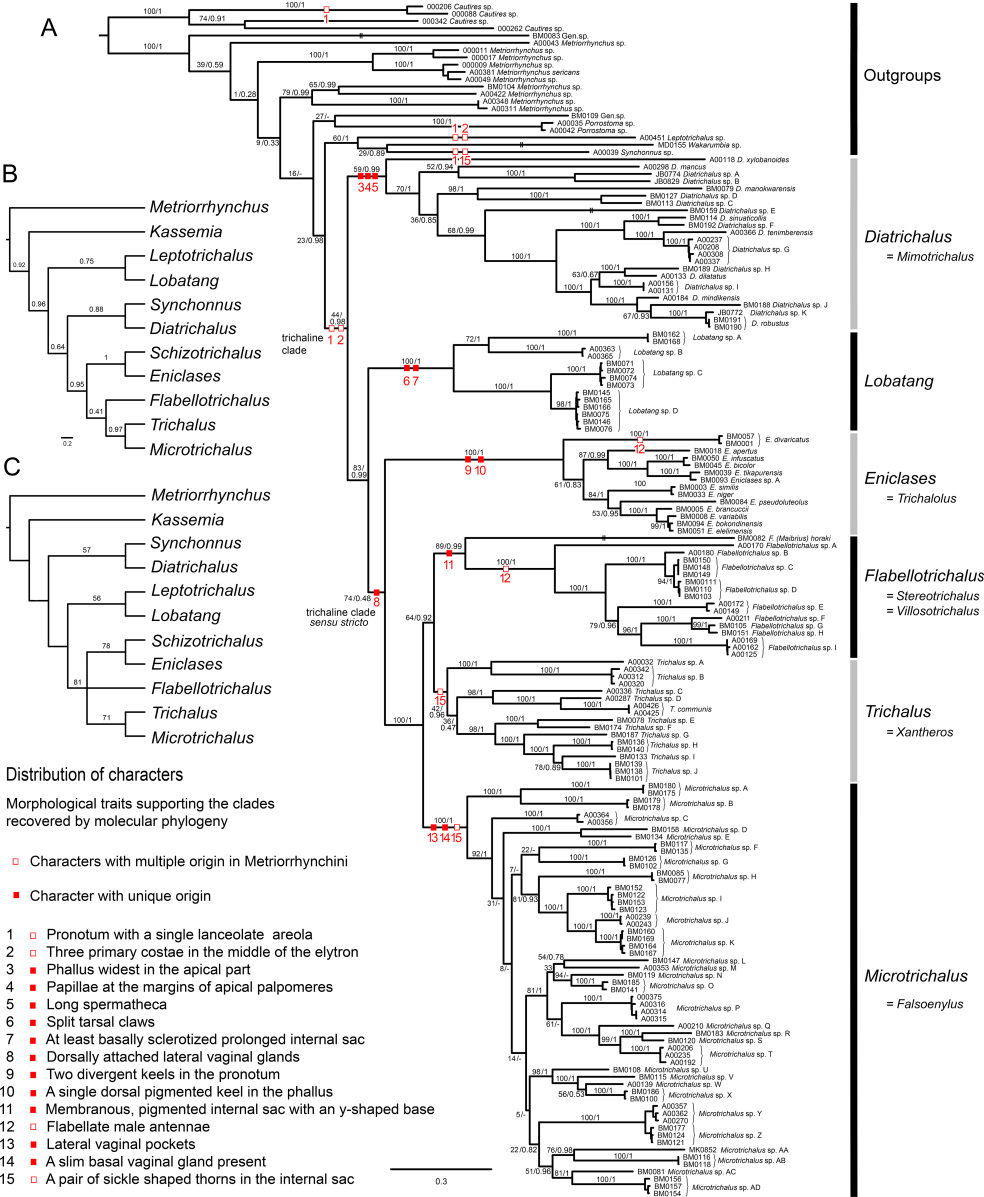
Phylogenetic hypotheses. (A) Molecular phylogenetic reconstruction of trichaline relationships using maximum-likelihood; (B) Bayesian phylogenetic reconstruction of trichaline morphological relationships, the maximum clade credibility tree with posterior probabilities mapped; (C) phylogenetic reconstruction of trichaline relationships inferred from morphology using the parsimony criterion. The topologies in B and C were inferred from morphological dataset shown in Table 1. The numbers at branches show bootstrap support values (A, values before slash and C) and posterior probabilities (A, values after slash, B). Only values over 50% shown in (C). Voucher numbers at branch tips identify the samples listed in Table 1.

The molecular dataset contained 143 ingroup terminals representing 86 species from the whole range of the trichaline clade. Three markers were sequenced: *rrnL* mtDNA (137 ingroup samples), *cox1–3′* end of mtDNA (137 samples), and *nad5* mtDNA (134 samples). The concatenated dataset consisted of 3,184 homologous positions: the alignments of the *rrnL*, *cox1*, and *nad5* fragments contained 808, 1,103, and 1,273 homologous base pairs, respectively. The phylogenetic trees inferred from the MAFFT alignment using the ML criterion and Bayesian inference were well-resolved and suggested similar relationships. The differences in the applied partitions and models proposed by PartitionFinder2 and jModelTest 2.1.7 did not have any effect on the ML topology and the bootstrap support values inferred in both analyses were highly similar and the topology is shown in Fig. 1A and Fig. S1. The differences reached up to 2% and can be explained by the stochastic character of bootstrap analyses. The results of analyses based on the jModelTest partitions and models are not shown and they are not discussed further. The BI topology differs only slightly in the outgroup and internal topology of the *Microtrichalus* clade (Fig. S1). However, ambiguities in hypothesized relationships within *Microtrichalus* were expected as all ML and BI analyses recovered low BS and PP values for most internal relationships (Fig. 1A; Fig. S1). The differences between analyses were limited to rearrangements in *Microtrichalus* clade and did not include relationships among genera (Fig. 1A). The trichaline clade was regularly recovered although only with an ambiguous support (BS 44%, PP 0.98). *Diatrichalus* marked the deepest node, followed by *Lobatang* and a clade of *Eniclases*, *Trichalus*, *Flabellotrichalus,* and *Microtrichalus*, further designated as the trichaline clade *sensu stricto. Schizotrichalus* was unavailable for molecular analyses. The genus-rank clades obtained mostly robust support >90% and regularly PP ∼1.0, except *Diatrichalus* (BS 59%, PP 0.99) and *Trichalus* (BS 42%, PP 0.96). The relationships among these deep nodes remain poorly supported. The sister clade of trichaline genera contains *Leptotrichalus*, *Synchonnus*, and *Wakarumbia* Bocak, 1999b.

### Morphological analysis

The morphological analyses did not support the monophyly of the DNA-based trichaline clade (Figs. 1B and 1C). The relationships of *Schizotrichalus*, *Eniclases*, *Flabellotrichalus*, *Microtrichalus*, and *Trichalus* were satisfactorily resolved only by the BI analysis (Fig. 1B; Fig. S2), but the MP analysis recovered three equally parsimonious trees (*L* = 38, CI = 0.737, RI = 0.714). Their strict consensus and one of the most parsimonious trees were unresolved (Fig. 1C). The deeper relationships were poorly supported. The only synapomorphy which confirms the monophyly of the (*Schizotrichalus*, *Eniclases*), *Flabellotrichalus*, (*Microtrichalus*, *Trichalus*) clade are the dorsally attached lateral vaginal glands (Figs. 1A and 6Q). The presence of thorns in the internal sac suggests relationships of *Trichalus* and *Microtrichalus* and the pigmented keel supports relationships of *Eniclases* + *Schizotrichalus*. All discussed character states, including apomorphies which support individual genera, are mapped on the molecular phylogeny in Fig. 1A.

### Taxonomy

#### Diagnosis of the trichaline clade

**Figure 2:**
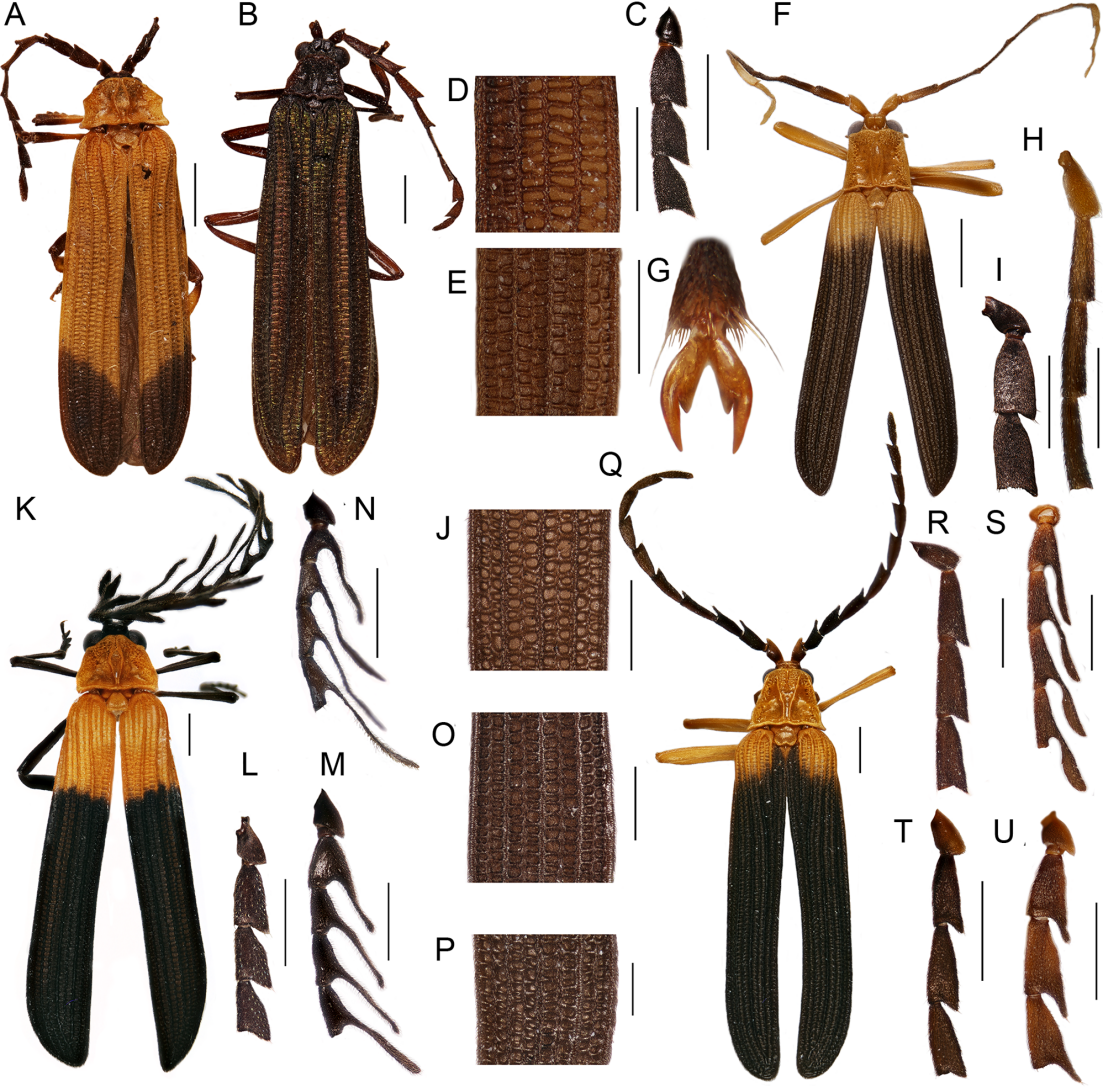
General appearance (1). General appearance, basal male antennomeres, and the posterior part of the right elytron. (A) *Diatrichalus* sp.; (B) *Diatrichalus aeneus* Bocak; (C) *Diatrichalus* sp.; (D) *D. cerberus* (Bourgeois), (E) *D. sinuaticollis* (Pic); (F) *Lobatang* sp.; (G) *L. papuensis* Bocak, hind tarsus claws; (H–J) *Lobatang* spp.; (K) *Flabellotrichalus* sp.; (L) *Flabellotrichalus* sp., female basal antennomeres, (M, N) *Flabellotrichalus* spp., male antennae; (O, P) *Flabellotrichalus* spp.; (Q) *Eniclases divaricatus* Kleine, female; *Eniclases* spp., male antennae: (R) *Eniclases* sp., (S) *E. divaricatus* Kleine; (T) *E. bicolor* Bocek et Bocak, (U) *E. similis* Bocak & Bocakova. Scales 1 mm (A, B, F, K, Q), 0.5 mm (other figures).

**Figure 3:**
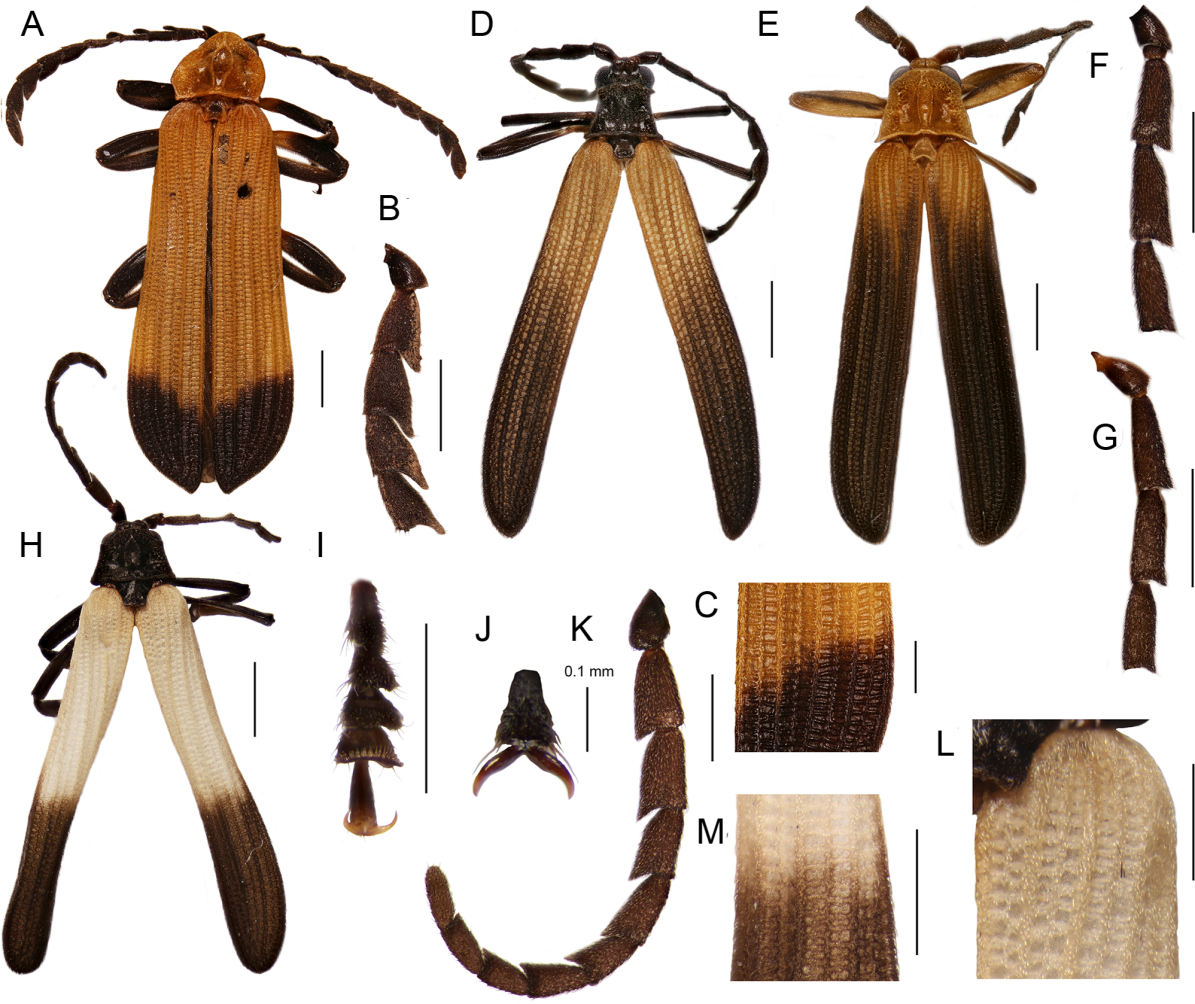
General appearance (2). General appearance, basal male antennomeres, and the posterior part of right elytron. (A–C) *Trichalus flavopictus*; (D) *Microtrichalus* sp., male; (E) *Microtrichalus* sp., female; (F, G) *Microtrichalus* spp.; *Flabellotrichalus* (*Maibrius*) *horaki* sp. nov.: (H) general appearance, (I) tarsus, (J) claws, (K) male antenna, (L) humeral part of elytron, (M) middle part of elytron. Scales 1 mm (A, D, E, H), 0.5 mm (B, C, F, G, I, K–M), 0.1 mm (J).

**Figure 4:**
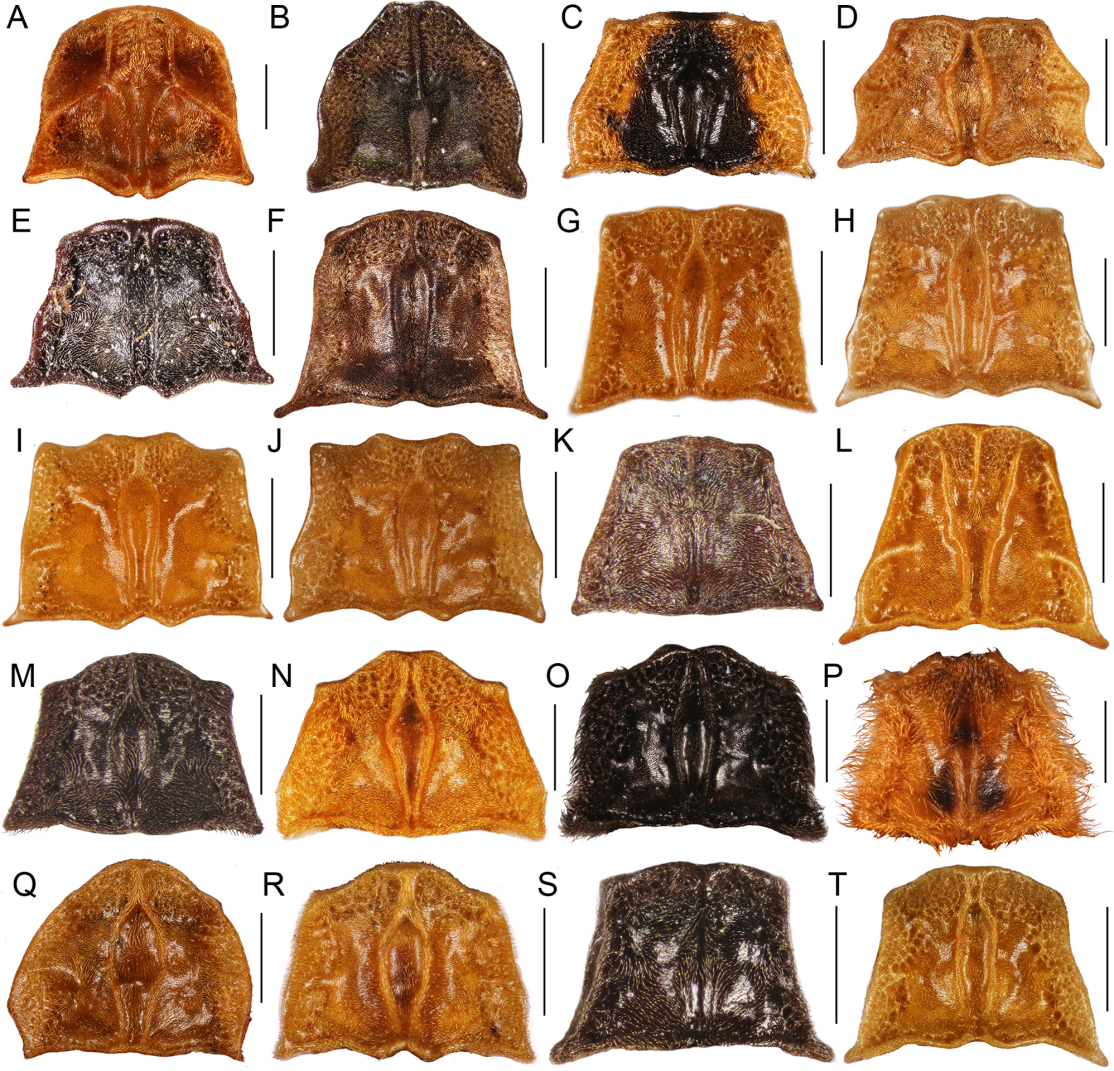
Pronota. (A) *Metriorrhynchus inaequalis* (F.); (B) *Bulenides* sp.; (C) *Diatrichalus* sp.; (D) *D. mancus* (Kleine); (E) *D. aeneus* Bocak; (F) *Lobatang papuensis* Bocak; (G–J) *Lobatang* spp.; (K) *Schizotrichalus* sp.; (L) *Eniclases divaricatus* Kleine; (M–P) *Flabellotrichalus* spp.; (Q) *Trichalus flavopictus* Waterhouse; (R) *T. communis* Waterhouse; (S, T) *Microtrichalus* spp. Scales 0.5 mm.

**Figure 5:**
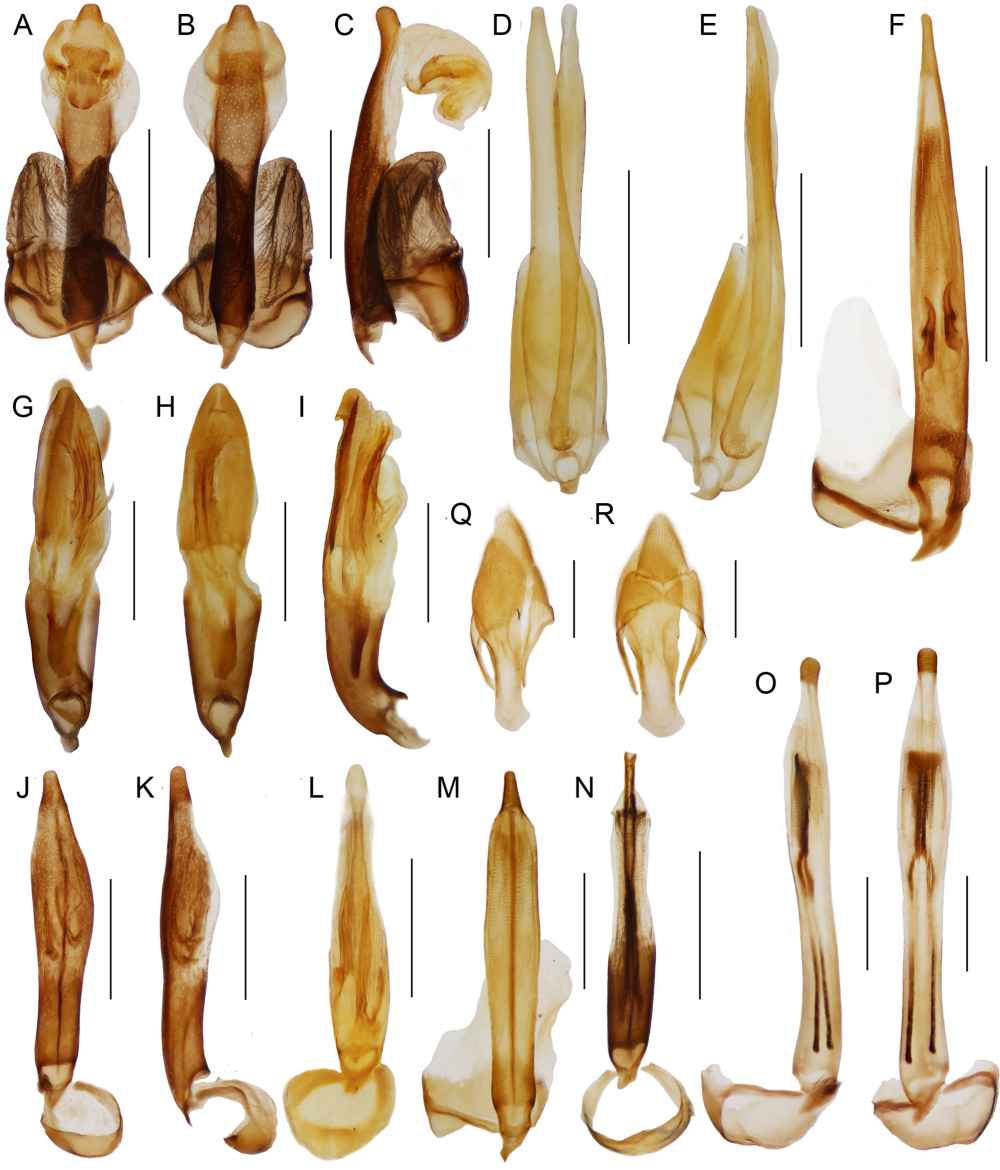
Male genitalia. Male genitalia and terminal abdominal sclerites. (A–C) *Diatrichalus* sp.; (D, E) *Lobatang* sp.; (F) *Trichalus flavopictus* Waterhouse; (G–I) *Lobatang* sp.; (J, K) *Trichalus* sp.; (L) *Microtrichalus* sp.; (M) *Eniclases* sp.; (N) *Flabellotrichalus* (*Maibrius*) *horaki* sp. nov.; (O, P) *Flabellotrichalus* sp.; (Q, R) *Lobatang* sp., male terminal abdominal sclerites, ventrally and dorsally. Scales 0.5 mm.

**Figure 6:**
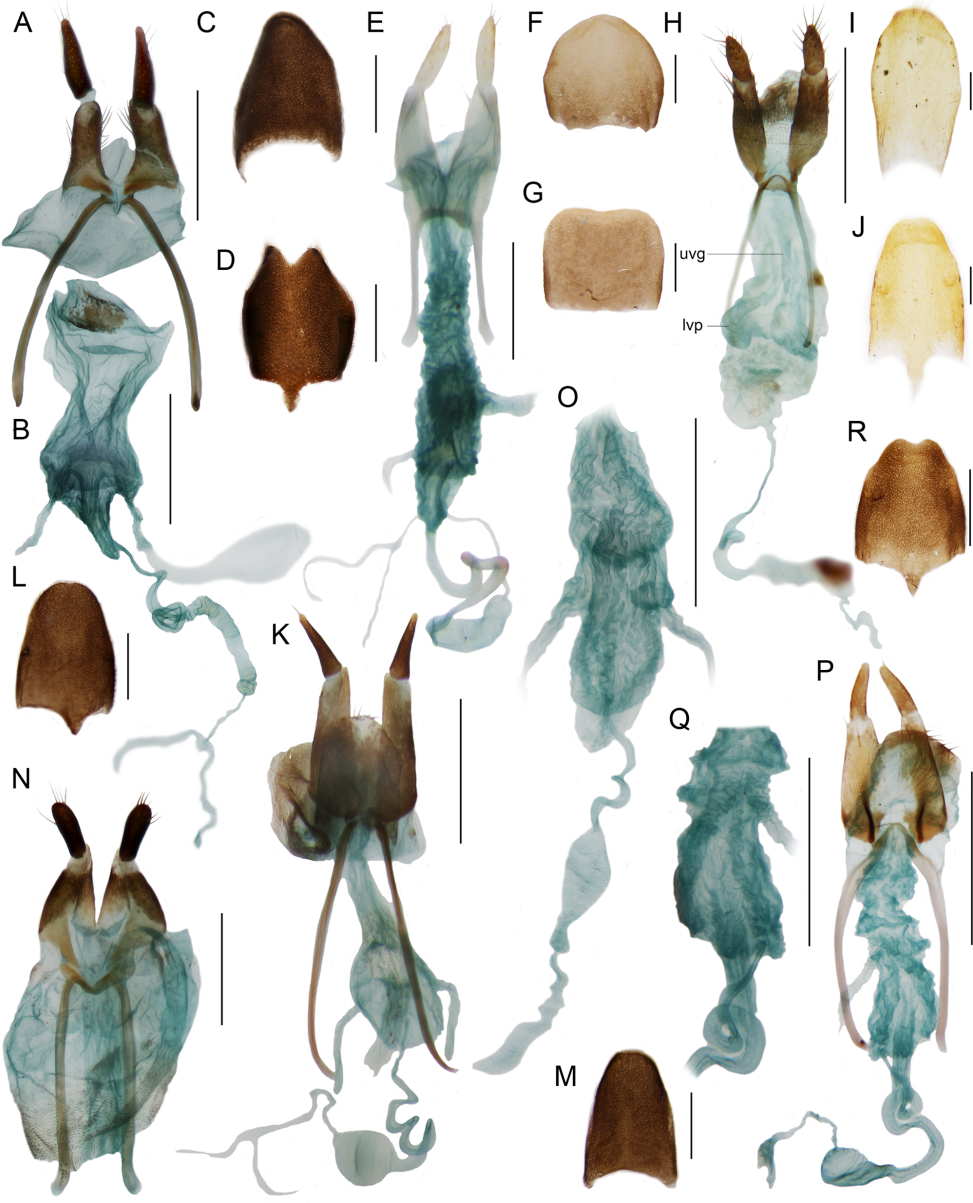
Female genitalia. Female genitalia and terminal abdominal sclerites. *Diatrichalus* sp. (A) ovipositor, (B) female genitalia, (C) terminal tergite, (D) terminal sternite; *Lobatang* sp. (E) ovipositor and female genitalia, (F) terminal sternite, (G) terminal tergite; *Microtrichalus* sp. (H) ovipositor and female genitalia, (I) terminal tergite, (J) terminal sternite; *Flabellotrichalus* sp. (K) ovipositor and female genitalia, (L) terminal sternite, (M) terminal tergite; *Trichalus* sp. (N) ovipositor, (O) female genitalia; *Eniclases divaricatus* Kleine (P) ovipositor, (Q) vagina, dorsally, (R) terminal sternite; uvg, unpaired gland; lvp, lateral vaginal pocket. Scales 0.5 mm.

Most trichaline genera may be distinguished from other Metriorrhynchini by their general appearance (Figs. 2 and 3) and external characters (Fig. 4). The pronotal carinae are reduced to a single, lanceolate areola in most genera (Figs. 4C–4J and 4M–4T); two divergent pronotal ridges are present in *Eniclases* and five areolae in *Schizotrichalus* (Figs. 4K and 4L). The first primary elytral costa is shortened in all trichaline genera (Figs. 2A, 2B, 2F, 2K, 2Q, 3A, 3D, 3E, 3H and 3L), and in some distantly related Metriorrhynchina, e.g., *Leptotrichalus* and *Kassemia* Bocak, 1998 (Bocak, 1998a, 2002). Male genitalia are highly variable, either robust with the characteristic sclerites in the internal sac (*Diatrichalus*; Figs. 5A–5C), the phallus is slender, with a simple sclerotized internal sac (*Lobatang*; Figs. 5D and 5E), robust with the sclerotized base of the internal sac (*Lobatang*; Figs. 5G–5I), slender with the mostly membranous internal sac with a pair of basal thorns (*Trichalus*, *Microtrichalus*; Figs. 5F and 5J–5L), slender with partly exposed, membranous internal sac (*Flabellotrichalus*; Figs. 5N–5P) or the phallus is almost completely membranous in the apical half and has a characteristic ventral pigmented keel and small cup-shaped apex (*Eniclases*, *Schizotrichalus*; Fig. 5M). The genital morphology of each genus is unique within Metriorrhynchini and enables reliable identification. Female genitalia have dorsally attached vaginal glands in *Schizotrichalus*, *Eniclases*, *Flabellotrichalus*, *Microtrichalus*, and *Trichalus* (Fig. 6Q), but the glands are laterally attached in *Diatrichalus* and *Lobatang* (Figs. 6B and 6E), as in other Metriorrhynchini.

Some trichaline net-winged beetles can be reliably identified only by a combination of characters. The pronotal carinae, elytral ridges and genitalia can be similar in distantly related metriorrhynchine taxa. Therefore, all these structures must simultaneously corroborate the membership in the trichaline clade.

### Redescription

Body small to medium-sized, 4–20 mm long, dorso-ventrally flattened, elytra parallel-sided or slightly widened backwards (e.g., Figs. 2A, 2B and 3A), body mostly dark brown, seldom yellow, upper side variably colored, often with aposematic color patterns combining yellow and dark colored parts; seldom some parts of pronotum and elytra brightly red colored or upper side metallic blue.

Head hypognathous, small, partly hidden by pronotum, rostrum absent in most species, sometimes moderately long rostrum in *Lobatang*. Cranium slightly dorso-ventrally flattened, with more or less prominent antennal tubercles followed by depression; mouth opening approximately as wide as long. Gula wider than long, with more or less wide process, where postmentum is attached; posterior tentorial pits usually unapparent externally; tentorium mostly membranous, only posterior tentorial arm partly sclerotized. Mandibles relatively stout, short, outer margin covered with dense long setae, sometimes only several short pale setae present. Labrum wider than long, shallowly emarginate apically, with long dense setae. Labium with robust praementum and much smaller u-shaped postmentum. Labial palpi with three palpomeres, palpomere 2 usually longest. Maxillae with long galea; lacinia smaller, sometimes reduced to limited field of pale short setae. Cardo very small, well-sclerotized, movable, stipes flat, with narrow bent inner margin. Maxillary palpi with four palpomeres, palpomeres 1 and 3 always much shorter than palpomeres 2 and 4. Apical palpomeres distally flattened. Antennae with 11 antennomeres, slightly to strongly flattened, antennomere 1 pear-shaped, robust, antennomere 2 very small, antennomeres 3–10 parallel-sided to acutely serrate in both sexes or flabellate in male and serrate in female, antennomere 11 elliptic; antennomeres 3–11 covered with dense, short pubescence.

Pronotum flat, with pronotal carinae (Figs. 4C–4T); *Diatrichalus, Lobatang, Flabellotrichalus*, *Trichalus,* and *Microtrichalus* with median lanceolate areola, *Eniclases* with two divergent longitudinal carinae (Fig. 4L), and *Schizotrichalus* with three areolae present within the area limited by longitudinal carinae (Fig. 4K). Median areola, if present, either connected with frontal margin by carina or attached directly to frontal and posterior pronotal margins, length of connecting carina variable; sometimes vestigial postero-lateral carinae present close to lateral margins (Figs. 4C–4T). Pronotal surface roughly punctured at frontal and lateral margins; pronotal pubescence usually short, sparse in most species, denser at lateral margins or very long and dense in some *Flabellotrichalus* (Figs. 4O and 4P). Prothoracic pleura concave, with strongly elevated margins, similarly structured as pronotal surface. Prothoracic coxal cavities open. Mesosternum transverse, narrow, bridge-like. Scutellum small, apex shallowly emarginate. Metathorax long, robust, metasternum broad and long, with incomplete midline in distal part.

Elytra flat, parallel-sided to slightly widened backwards, each elytron with nine longitudinal costae at base; four costae robust, called primary costae, intermediate secondary costae weak, sometimes irregular. Primary costa 1 robust only in humeral quarter of elytron, then much weaker, similar to secondary costae; secondary costae between suture and primary costa 1 and between primary costae 1 and 2 missing except humeral quarter of elytron (Figs. 3A, 3D, 3E and 3L); seldom secondary costae absent (some *Diatrichalus*; Fig. 2D).

Abdomen flat, free, with eight visible sternites in male and seven in female. Shape of male terminal sternites variable, affected by shape of phallus. Subapical male abdominal sternite more or less emarginate at hind margin. Last visible tergite long, spoon-like, often with small sclerotized tergite attached to inner surface, this tergite sometimes membranous, undetectable. Female terminal abdominal segments variable in shape and most species with short spiculum gastrale (Figs. 6C, 6D, 6I, 6J, 6L, 6M and 6R).

Male genitalia variable in shape (Figs. 5A–5P). Phallobase circular, subtle, with more or less extensive membrane, membrane soft to lightly sclerotized. Parameres absent, phallus mostly slender, with well-sclerotized or partly membranous apical part, open ventrally with exposed internal sac. Internal sac membranous to sclerotized, with apical complex sclerite or with pair of slender sickle-like thorns at base.

Ovipositor mostly with long, slender valvifers (Figs. 6A, 6H, 6K, 6O and 6P), sometimes valvifers connected at their bases by membrane, which can be sclerotized in high degree; seldom valvifers basally fused with coxites (Fig. 6E). Valvifers robust, connected in basal third in some *Trichalus*. Vagina slender, paired glands inserted laterally (Fig. 6B) or dorsally (Fig. 6Q). Bases of glandular ducts slender, seldom robust (*Trichalus*), but regularly more sclerotized than terminal gland, flat unpaired gland in terminal part of vagina, lateral pockets and slender unpaired basal gland in *Microtrichalus* (Fig. 6H). Spermatheca long, and slender (Fig. 6B), lemon-shaped, with spirally coiled spermaduct; y-shaped gland attached to apex of spermatheca (Fig. 6K).

***Diatrichalus*Kleine, 1926**
(Figs. 2A–2E, 4C–4E, 5A–5C and 6A–6D)
*Diatrichalus* Kleine, 1926: 167.

**Type species:** *Diatrichalus xylobanoides* Kleine, 1926, by original designation.

=*Mimotrichalus* Pic, 1930: 92, hors texte; Bocak, 1998a: 182.

**Type species:** *Mimotrichalus tenimberensis* Pic, 1930, by monotypy.

**Diagnosis:** Pronotum with median, often wide areola, lateral carinae absent or very obtuse (Figs. 4C–4E), antennae of both sexes more or less acutely serrate to shortly flabellate (Fig. 2C), phallus stout, apical part projected, internal sac more or less sclerotized (Figs. 5A–5C), vaginal glands inserted laterally, valvifers free, slender, spermatheca long, slim (Figs. 6A and 6B), tarsal claws simple.

**Remark:** Kleine (1926) restricted *Diatrichalus* to species with four elytral costae, as in *D. xylobanoides* (Fig. 2D), and Pic described *Mimotrichalus* as having additionally obtuse, irregular and commonly interrupted secondary costae. The current concept of *Diatrichalus* is wide and includes all species with four and nine costae and their intermediate forms (Figs. 1A, 2D and 2E; Bocak, 2001). Our molecular dataset contained only a single species without secondary elytral costae, *D. xylobanoides*, which is a sister species to other *Diatrichalus*, included in the analyses. The current results support two clades which correspond with earlier concepts of *Diatrichalus* and *Mimotrichalus*, but Bocak (2001) showed that other species without secondary costae have diverse genitalia, and we suppose that if these are included in future phylogenetic analyses they will not form a monophylum. Additionally, there are multiple species with gradual reduction of secondary costae and they can only be arbitrarily assigned to their respective groups. Therefore, we propose to keep *Mimotrichalus* in the synonymy of *Diatrichalus*. Although the antennae have never long lamellae, they are sometimes so acutely serrate that Kleine (1933b) classified *D. salomonensis* (Kleine, 1933b) in *Flabellotrichalus* (Bocak, 2001).

***Lobatang*Bocak, 1998a**
(Figs. 2F–2I, 4F–4J, 5D, 5E, 5G–5I and 6E–6G)
*Lobatang* Bocak, 1998a: 190.

**Type species:** *Lobatang papuensis* Bocak, 1998a.

**Diagnosis:** Antennomeres 3–10 parallel-sided to serrate (Figs. 2H and 2I), pronotum with median lanceolate areola (Figs. 4F–4J), male genitalia variable in shape, always with sclerotized base of internal sac (Figs. 5G–5I) or whole internal sac sclerotized and long (Figs. 5D and 5E), tarsal claws split (Fig. 2G).

**Remark:** The clade *Leptotrichalus* + *Lobatang* was based on the shape of valvifers (Bocak, 1998a, 2002), but the molecular phylogeny indicates the distant position of these genera (Fig. 1A; Sklenarova, Kubecek & Bocak, 2014).

***Lobatang* s. str.**

**Type species:** *Lobatang papuensis* Bocak, 1998a.

**Diagnosis:** The nominotypical subgenus differs from *Spinotrichalus* only in the absence of femoral and tibial thorns in hind legs.

**Subgenus *Spinotrichalus*Kazantsev, 2010, stat. nov.**
*Spinotrichalus* Kazantsev, 2010: 93.

**Type species:** *Spinotrichalus telnovi* Kazantsev, 2010, by original designation.

**Diagnosis:** As the nominotypical subgenus, but hind femora and tibiae with small thorns.

**Remark:** Kazantsev (2010) described *Spinotrichalus*, which shares very similarly shaped genitalia and split claws with *Lobatang*. Besides the body shape and coloration, the type species of *Spinotrichalus* and *Lobatang* differ only in the presence of femoral and tibial thorns. This character is the autapomorphy of *S. telnovi* and *Spinotrichalus* may be treated as a synonym, if its position renders *Lobatang* paraphyletic. As the type species of both genera are unavailable for DNA analysis, we prefer to keep *Spinotrichalus* as a valid name till more data are available. Based on highly similar male genitalia (Figs. 5D and 5E; Kazantsev, 2010), we lower its rank to a subgenus of *Lobatang* Bocak, 1998a. Consequently, the new combination *Lobatang* (*Spinotrichalus*) *telnovi* (Kazantsev, 2010) is proposed.

***Eniclases*Waterhouse, 1879**
(Figs. 2Q–2U, 4L, 5M, 6P and 6R)
*Eniclases* Waterhouse, 1879: 66.

**Type species:** *Lycus* (genus 35) *luteolus* Waterhouse, 1878, by original designation.

=*Trichalolus* Pic, 1923: 36, hors texte; Bocak & Bocakova, 1991: 206.

**Type species:** *T. apertus* Pic, 1923, by monotypy.

**Diagnosis:** Pronotum with two longitudinal divergent carinae dividing pronotum in three fields (Fig. 4L), phallus very slender with pigmented dorsal keel, internal sac without thorns; whole internal sac membranous (Fig. 5M); lateral vaginal glands dorsally attached (as in Fig. 6Q).

**Remark:** The *Eniclases* male antennae are highly variable in shape and several species have acutely serrate to flabellate antennae (Figs. 2R–2U; Bocak & Bocakova, 1991; Bocek & Bocak, 2016). Only one of these species was included in the molecular analysis and it was recovered as a sister to its congeners (Fig. 1A). Other morphological characters and molecular phylogeny indicate that the species with similar antennae are not closely related (Bocek & Bocak, 2016; Bocak & Bocakova, 1991). Therefore, we do not consider this character to be valuable in the delimitation of a genus or subgenus in this clade.

***Schizotrichalus*Kleine, 1926**
(Fig. 4K)
*Schizotrichalus* Kleine, 1926: 167.

**Type species:** *T. nigrescens* Waterhouse, 1879, by original designation.

**Diagnosis:** Pronotum with five areolae (Fig. 4K), phallus with pigmented dorsal keel, internal sac without thorns; vaginal lateral glands dorsally attached.

**Remark:** *Schizotrichalus* was unavailable for molecular analyses and was inferred as a genus closely related to *Eniclases* in the morphology-based phylogeny (Figs. 1B and 1C; Bocak, 1998a, 2002).

***Flabellotrichalus*Pic, 1921b**
(Figs. 2K–2P, 3H–3M, 4M–4P, 5N–5P and 6K–6M)
*Flabellotrichalus* Pic, 1921b: 9, hors texte.

**Type species:** *Flabellotrichalus notatithorax* Pic, 1921, subsequent designation, Kleine (1936).

*=Stereotrichalus* Kleine, 1926: 183; Kleine, 1930: 330.

**Type species:** *Stereotrichalus evidens* Kleine, 1926, by monotypy.

*=Villosotrichalus* Pic, 1921b: 9, hors texte; Bocak, 1998a: 183.

**Type species:** *Villosotrichalus reductus* Pic, 1921b, by monotypy.

**Diagnosis:** Male antennae flabellate (Figs. 2M and 2N) or seldom serrate (Fig. 3K), pronotum with single longitudinal median areola, frontal and lateral margins of pronotum often with dense short to very long pubescence (Figs. 4M–4P), phallus very slender, internal sac without thorns; whole internal sac membranous with y-shaped base (Figs. 5N–5P); lateral vaginal glands attached dorsally.

**Remark:** The molecular phylogeny recovered a species with dense pronotal pubescence in the terminal position (Fig. 1A) which supports the earlier synonymization of *Villosotrichalus* to *Flabellotrichalus* (Bocak, 1998a).

**Subgenus *Flabellotrichalus*Pic, 1921b**

**Diagnosis:** All diagnostic characters as in the whole genus, but the male antennae are always flabellate (Figs. 2M and 2N).

**Classification and distribution:** *Flabellotrichalus* occur in Australia, New Guinea, and the Moluccas. Nine Australian and New Guinean species were included in current analyses, but none was identified to the species level due to chaotic alpha-taxonomy (Fig. 1). The genus has never been revised and all 15 formally described species are known only from original descriptions. Two species with dense pronotal pubescence were classified originally as *Villosotrichalus* and this genus was synonymized with *Flabellotrichalus* (Bocak, 1998). The species similar to the typical *Villosotrichalus* were inferred in the terminal position within *Flabellotrichalus* in current analyses (Fig. 1A).

**Subgenus *Maibrius* subgen. nov.**
LSID: urn:lsid:zoobank.org:act:0A2E45FB-72DB-49E7-BD7C-BC792072B106
(Figs. 3H–3M, 4M and 5N)

**Type species:** *Flabellotrichalus* (*Maibrius*) *horaki* sp. nov.

**Diagnosis:** Male antennae serrate (Fig. 3K), pronotum with single longitudinal median areola, frontal and lateral margins of pronotum with dense short pubescence (Fig. 4M), phallus slender, apically membranous; internal sac without thorns, membranous, with y-shaped base (Fig. 5N); lateral vaginal glands attached dorsally. *Maibrius* subgen. nov. differs from the nominotypical subgenus in the serrate male antennae (Fig. 3K) and shorter, relatively robust phallus (Fig. 5N).

**Remark:** The molecular phylogeny identified *F.* (*Maibrius*) *horaki* sp. nov. as a genetically distant sister-lineage to other *Flabellotrichalus* (Fig. 1A). This species cannot be identified as a close relative of *Flabellotrichalus* without dissection of male genitalia or DNA sequencing. The general appearance and morphology of antennae resemble *Trichalus* or *Microtrichalus* and only the male genitalia indicate relationships to *Flabellotrichalus*. This conservative taxonomy keeps *Flabellotrichalus* s. str. morphologically well-defined and reflects the genetic and phenotypic divergence of *F*. (*Maibrius*) *horaki* sp. nov. Female remains unknown.

**Etymology:** The subgeneric name is derived from the name “Maibri,” a village in the Arfak mountains where the type species was collected. The genus name is the noun of masculine gender.

***Flabellotrichalus* (*Maibrius*) *horaki* sp. nov.**
LSID: urn:lsid:zoobank.org:act:86069ACA-BC85-4865-847B-2EB421DC3BC3
(Figs. 3H–3M, 4M and 5N)

**Type material:** Holotype. Male, “New Guinea, West Papua prov., Arfak Mts., Maibri vill., 2015, local coll.” (GenBank Voucher Number UPOL BM0082; deposited in the collection of the Palacky University in Olomouc, Czech Republic, LMBC).

**Diagnosis:** *Flabellotrichalus* (*Maibrius*) *horaki* sp. nov. differs from all known *Flabellotrichalus* in the serrate male antennae (Fig. 3K). Its phallus is slightly more robust than in other *Flabellotrichalus* (Figs. 5N–5P). *F.* (*M.*) *horaki* sp. nov. is currently a single trichaline species with white colored humeri.

**Description:** Male. Body 7.8 mm long, dorso-ventrally flattened, relatively slender, dark brown to black, only basal three fifths of elytra pale yellow to white colored (Fig. 3H). Head small, eyes small-sized, hemispherically prominent, eye diameter 0.64 times interocular distance; antennae serrate (Fig. 3K). Pronotum 1.24 wider than long at midline, trapezoidal, widest at base, anterior angles almost rectangular, well-marked, lateral margins slightly concave, posterior angles sharply prominent; areola wide, connected with anterior margin by short carina, lateral carinae completely absent, disc of pronotum roughly sculptured at frontal and lateral margins, covered with dense, short pubescence (Fig. 4M). Elytra with three primary and four secondary costae in middle part of elytron, elytra 3.7 times longer than width at humeri, rectangular cells dense, irregular, costae covered with dense pubescence (Figs. 3L and 3M). Phallus relatively short, sclerotized and pigmented in basal two fifths, apical part membranous, with a cup-shaped apex held by pair of pigmented keels; internal sac membranous, with y-shaped, pigmented base, without any thorns (Fig. 5N). Legs flattened, densely pubescent, tarsi wide (Fig. 3I), claws simple (Fig. 3J). Female unknown.

**Measurements:** Body length 7.8 mm, pronotum length 0.91 mm, pronotum width 1.13 mm, width at humeri 1.75 mm, length of elytron 6.55 mm, eye diameter 0.38 mm, eye distance 0.59 mm, length of phallus 1.14 mm.

**Etymology:** The specific name is a patronym in honor of Jan Horak, a Czech specialist in Mordellidae.

**Distribution:** New Guinea, Arfak mountains.

***Trichalus*Waterhouse, 1877**
(Figs. 3A–3C, 4Q, 4R, 5F, 6N and 6O)
*Trichalus* Waterhouse, 1877: 82.

**Type species:** *T. flavopictus* Waterhouse, 1877, subsequent designation, Waterhouse, 1878: 103.

*=Xantheros* Fairmaire, 1877: 167; Bourgeois, 1891: 347.

**Type species:** *Xantheros ochreatus* Fairmaire, 1877.

**Diagnosis:** Antennae serrate in both sexes, pronotum with single longitudinal median areola, apical part of phallus commonly well-sclerotized (Figs. 5F, 5J and 5K), internal sac with two thorns; lateral vaginal glands attached dorsally, valvifers free or connected basally (Fig. 6N) or sub-basally, forming H-shaped structure in some species, tarsal claws simple, vaginal lateral pockets and unpaired basal gland absent.

**Remark:** The type *of X. ochreatus*, the type species of *Xantheros*, was very probably destroyed (Bocak, 1998a). The original publication cites “Sydney” as the type locality and although we had at our disposal the extensive collection of Australian trichaline net-winged beetles from ANIC (Canberra), we found no specimen whose morphology agrees to the original description and originates from southern New South Wales. Similar species occur only in northern New South Wales and in Queensland. As we are not able to designate the neotype, we keep *Xantheros* in synonymy of *Trichalus* (Kleine, 1933a; Bocak, 1998a, 2002).

***Microtrichalus*Pic, 1921b**
(Figs. 3D–3G, 4S, 4T, 5L and 6H–6J)
*Microtrichalus* Pic, 1921b: 9 (hors texte).

**Type species:** *M. singularis* Pic, 1921b, by monotypy.

*=Falsoenylus* Pic, 1926: 29, hors texte; Bocak, 1998a: 184.

**Type species:** *F. basipennis* Pic, 1926, by monotypy.

**Diagnosis:** Antennae weakly serrate in both sexes, pronotum with single longitudinal median areola, apical part of phallus weakly sclerotized, internal sac with two thorns, lateral vaginal glands attached dorsally, vagina with two lateral pockets situated in middle of vaginal length and very slim, long, unpaired gland between valvifers (Fig. 6H), valvifers slender, sometimes fused basally.

**Key to the genera and subgenera of the trichaline clade**
1. Tarsal claws split (Fig. 2G), *Lobatang* Bocak, 1998a2
  —Tarsal claws simple (Fig. 3J)3
2. Male hind femora and tibiae without any thorn***Lobatang*** (***Lobatang*** s. str.)
  —Male hind femora and tibiae with small thorns***Lobatang*** (***Spinotrichalus*** Kazantsev, 2010)
3. Apical margins of maxillary and labial palpomeres with sensillae, apical palpomeres securiform, apical part of phallus robust, internal sac complex, partly sclerotized (Figs. 5A–5C); vaginal glands inserted laterally (Fig. 6B), basal part of spermaduct wide, spermatheca long, slender***Diatrichalus*** Kleine, 1926
  —Apical margins of maxillary and labial palpomeres without sensillae, apical palpomeres variable shaped, apical part of phallus slender, internal sac membranous or with a pair of sickle shaped thorns (Figs. 5F and 5J–5P); vaginal glands inserted dorsally (Fig. 6Q), basal part of spermaduct slender, spermatheca bulbous (Figs. 6H, 6K, 6O and 6P)4
4. Pronotum with five areolae or with two anteriorly divergent longitudinal carinae (Figs. 4K and 4L), phallus with single pigmented dorsal keel (Fig. 5M)5
  —Pronotum with single lanceolate longitudinal areola attached to frontal and basal margin of pronotum at a single point (Figs. 4M–4T), pigmented dorsal keel absent in most species (Figs. 5F and 5N–5P, but compare with Fig. 5J)6
5. Pronotum with five areolae (Fig. 4K)***Schizotrichalus*** Kleine, 1926
  —Pronotum with two divergent longitudinal carinae (Fig. 4L)***Eniclases*** Waterhouse, 1879
6. Male antennae flabellate***Flabellotrichalus*** (***Flabellotrichalus*** s. str.)
  —Male antennae serrate or antennomeres parallel-sided7
7. Internal sac membranous, without thorns, with pigmented y-shaped basal part (*Maibrius* females are unknown)***Flabellotrichalus*** (***Maibrius* subgen. nov.**)
  —Internals sac with two thorns8
8. Vagina with two lateral pockets in middle part and with unpaired slim and long basal gland (Fig. 6H), valvifers slender, usually free, sometimes connected basally***Microtrichalus*** Pic, 1921b
  —Vagina without lateral pockets and unpaired gland, valvifers often robust, connected basally or sub-basally (Fig. 6N)***Trichalus*** Waterhouse, 1877

## Discussion

We present the first densely sampled molecular phylogeny and separate morphological analyses of all genera which were traditionally placed in the trichaline clade (Figs. 1A–1C). The terminal position of the trichaline clade in Metriorrhynchina has already been demonstrated in the molecular analyses of Metriorrhynchini, and trichaline genera lost their formal rank in classification (Sklenarova, Kubecek & Bocak, 2014). Our analyses of the current more extensive dataset confirm the terminal placement of the trichaline clade within Metriorrhynchina (Fig. 1A). Metriorrhynchina are well-supported as a monophylum in all previous analyses (Bocak et al., 2008; Sklenarova, Chesters & Bocak, 2013; Sklenarova, Kubecek & Bocak, 2014), therefore, Cautirina were used as an outgroups and Metriorrhynchina, here consisting of trichaline terminals and 17 non-trichaline terminals, were forced by a single outgroup to be monophyletic. Such dataset is fully capable to test if trichaline genera are a sister lineage of other Metriorrhynchina or a terminal lineage within this subtribe as in all earlier analyses (Bocak et al., 2008; Sklenarova, Chesters & Bocak, 2013; Sklenarova, Kubecek & Bocak, 2014).

The (*Leptotrichalus*, (*Synchonnus*, *Wakarumbia*)) clade is a sister lineage to trichaline genera in the molecular analyses although with ambiguous support (BS 23%; PP 0.98; Fig. 1A). *Leptotrichalus* and *Synchonnus* were earlier placed in the trichaline clade, but *Wakarumbia* differs substantially in the presence of unique five-areolae in the pronotum, full-length elytral costae, and the morphology of genitalia (Bocak, 2002). Therefore, an expansion of the trichaline clade would be impractical.

Four trichaline genera are included in our molecular analyses for the first time and now six of seven genera are represented in the DNA data set: *Diatrichalus* and *Lobatang* are members of the trichaline clade as defined here and they are deeply rooted lineages in close relationships to the earlier narrowly defined trichaline clade (Bocak, 1998a, 2002). *Eniclases* is a sister to the clade ((*Flabellotrichalus*, *Trichalus*), *Microtrichalus*) (Fig. 1A).

The morphological analyses indicate different relationships. They suggest a topology which contains the clades (*Synchonnus + Diatrichalus*) and (*Leptotrichalus + Lobatang*) in contrast with molecular analyses (Fig. 1A; Sklenarova, Kubecek & Bocak, 2014). Such relationships are supported by the similar shape of pronotal carinae in trichaline genera, *Synchonnus*, and *Leptotrichalus* and the shortened elytral costa 1 in all genera except *Synchonnus*. Due to the limited number of other informative phenotypic characters, the homology of these character states cannot be falsified in the current morphological analyses (Figs. 1A–1C). The single lanceolate areola and the shortened elytral costa 1 were present in the most recent common ancestor of the trichaline clade (Fig. 1A), but similar arrangements of pronotal carinae and elytral costae have been found in several unrelated taxa, e.g., the shortened costa in *Kassemia* and the similar pronotum in some *Cautires* (Bocak, 2002; Sklenarova, Kubecek & Bocak, 2014). The high plasticity of pronotal carinae is additionally indicated by a hypothesized reversal in *Eniclases* and *Schizotrichalus* (Fig. 1A). Therefore, we consider the phylogenetic signal provided by these external characters to be unreliable and male and female genitalia should be studied to verify recovered relationships.

The molecular topology regularly indicates a deep position of *Diatrichalus* and *Lobatang*, but we have not been able to find any phenotypic character which supports their relationships with other trichaline genera, except for the above mentioned lanceolate pronotal areola and the shortened elytral costa 1. Conversely, the monophyly of the restricted trichaline clade, i.e., *Eniclases* + *Flabellotrichalus* + *Trichalus* + *Microtrichalus* is supported by unique, dorsally attached vaginal glands (Fig. 6Q) in the morphological analysis, but their relationships, although simultaneously recovered by molecular analyses, had only a low statistical support (BS 74%, PP 0.48). The internal relationships within this clade were better resolved in the DNA-based topology, which indicates the deeply rooted position for *Eniclases* with respect to other genera of the restricted trichaline clade (Figs. 1A–1C). *Schizotrichalus* was not available for the molecular analyses and its close relationships with *Eniclases* are based on morphology (Figs. 1B and 1C). *Trichalus* and *Flabellotrichalus* form a clade with a low support in molecular analyses (BS 64%, 0.92 PP) and their sister position has never been inferred from morphology (Figs. 1A–1C). Their relationship is supported by similar pigmented keels at the apex of the phallus in some species, but no other character (Figs. 5F and 5N–5P). In contrast, *Microtrichalus* and *Trichalus* share sickle-shaped thorns in the basal part of their internal sac (Figs. 5F and 5J–5L). Concerning the low bootstrap support, these relationships need further data to be validated. Additionally, *Trichalus* is not assuredly monophyletic (Fig. 1A) and may split into several clades if more taxa are included in future analyses. The absence of a synapomorphy which supports *Trichalus* also complicates identification. Some species cannot be reliably identified as *Trichalus* without information on female genitalia. *Microtrichalus* has unique pockets in the middle part of the vagina and an unpaired basal vaginal gland (Fig. 6H). Both structures are absent in *Trichalus*.

For a long time, the phenotypic diagnoses of most trichaline genera were ambiguous. *Trichalus* served as a basket where most species were placed, and numerous species were later transferred to *Diatrichalus*, *Lobatang*, and *Microtrichalus* (Kleine, 1926; Bocak, 1998a, 2000, 2001). Now, the generic limits are much better defined than in the original descriptions and concepts applied by M. Pic and R. Kleine (Kleine, 1926; Pic, 1921b, 1923, 1926, 1930), but even with these revised morphological diagnoses, the evaluation of external phenotypic characters is generally insufficient and dissection of genitalia is needed for reliable generic placement.

Some phenotypic characters are affected by the natural and sexual selection and they can rapidly evolve (Bocek & Bocak, 2016; Frazee & Masly, 2015). Hence, they may provide a misleading phylogenetic signal. Below, we discuss some characters with regard to their diagnostic value and congruence with molecular phylogeny.

### The shape of male antennae

Filiform, serrate and flabellate male antennae have been used as diagnostic characters, but their value is questioned by variable morphology in related species (e.g., *Cautires*; Sklenarova, Kubecek & Bocak, 2014). A high variability in the shape of male antennae was observed in *Lobatang* (Figs. 2H and 2I) and *Eniclases* (Figs. 2R–2U); other genera, such as *Microtrichalus*, have quite uniform antennae (Figs. 3F and 3G). The present study supports the earlier finding that the serrate and flabellate antennae can evolve repeatedly. *Diatrichalus salomonensis* (Kleine, 1933b) and some species of *Eniclases* (Figs. 1A and 2R–2U) have very acutely serrate to flabellate antennae, unlike the congeneric species. *Flabellotrichalus* s. str. is well-delimited by the flabellate antennae. We identified a single species, *F.* (*Maibrius*) *horaki* sp. nov., which differs in the serrate male antennae and is also genetically distant from other *Flabellotrichalus.* It was recovered as a sister to the extensive clade of *Flabellotrichalus* s. str. The antennae are an olfactory organ and selection for a large surface can be responsible for rapid morphological evolution in some terminal lineages.

### The shape of the pronotum and pronotal carinae

The shape of the pronotum is commonly used for morphological identification of net-winged beetle genera and some trichaline species can be assigned to a genus using pronotal morphology. The densely pubescent pronotal margins are characteristic for some but not all *Flabellotrichalus* (Figs. 4O and 4P). Transverse pronota with a large median areola and uniquely shaped lateral margins are characteristic for some *Diatrichalus* (Fig. 4D), but these traits are inconspicuous in some congeneric species (Figs. 4C and 4E). Similarly, the flat pronotum with the characteristic shape of the frontal margin and almost rectangular anterior angles is typical of some, but not all, *Lobatang* (Figs. 4F–4J). The shape of the pronotum is affected by the general appearance (e.g., Figs. 3D and 3E). Net-winged beetles are often associated with mimicry rings and substantially different body sizes, shapes and colorations were identified in recently split sister species, e.g., in *Eniclases* and *Synchonnus* (Bocek & Bocak, 2016; Kusy, Sklenarova & Bocak, in press). Therefore, these characters, although sometimes useful for quick identification, are generally unreliable, as can be demonstrated by similar pronota in several species of *Lobatang* (Fig. 4F), *Flabellotrichalus* (Fig. 4M), *Trichalus* (Fig. 4R), and *Microtrichalus* (Figs. 4S and 4T).

An earlier study has already demonstrated that the unique arrangement of seven pronotal areoles is an ancestral state in Metriorrhynchina (Fig. 4A; Sklenarova, Kubecek & Bocak, 2014). Although numerous species have the full number of seven areoles (Fig. 4A; *Cautires*, *Metriorrhynchus* Gemminger & Harold, 1869, *Porrostoma* Castelnau, 1838, and others) or their reduction is so limited that the original pattern can easily be recognized (some *Cautires*; Jiruskova, Motyka & Bocak, 2016), there are numerous genera with considerably simplified pronotal carinae. When these reduced patterns are considered to be homologous, they lead to a false phylogenetic placement and classification, as occurred when the monophyly was hypothesized and the genus-rank given to *Bulenides*, now placed in *Cautires* (Fig. 4B; Dudkova & Bocak, 2010) and also when an independent position and high rank were proposed for trichaline genera (Kleine, 1928, 1933a; Bocak, 1998a, 2002). The earlier defined family rank taxon for trichaline genera, including *Leptotrichalus* (Kleine, 1928, 1933a), was defined by a single areola in most genera: the wide areola in *Diatrichalus* (Figs. 4C and 4D), the very slender areola in *Leptotrichalus*, and a single narrow areola in *Microtrichalus* and *Trichalus* (Figs. 4Q–4T). A similar single areola has been identified in distantly related net-winged beetles, such as Afrotropical Slipinskiini, which had been considered congeneric with the Australian metriorrhynchine genus *Stadenus* Waterhouse, 1879 (Kleine, 1933a). Similarly, the arrangement of pronotal carinae in some *Synchonnus*, a genus related to *Falsolucidota* Pic, 1921a and *Wakarumbia*, provided a misleading signal for the placement of an earlier valid *Enylus* into close relationships with the trichaline genera (Figs. 1B and 1C; Bocak, 2002; Kusy, Sklenarova & Bocak, in press). The complex structures are considered to be better indicators of relationships, but in the case of *Eniclases* and *Schizotrichalus*, unique characteristic pronotal patterns, apparently resembling the complex ancestral arrangement (Figs. 4K and 4L), were recovered in the terminal lineage of the trichaline clade in which all close relatives lost the fronto-lateral pronotal carinae (Figs. 1A–1C and 4A–4T). Our results suggest that variable arrangements of pronotal carinae can evolve through reductions in unrelated lineages and, surprisingly, also through the re-appearance of earlier lost structures. These facts indicate the low explanatory power of this character for phylogenetic inference and generic classification (Fig. 1A).

### Elytral costae

Elytral costae were traditionally considered to be reliable characters for generic phenotypic diagnoses in net-winged beetles (Pic, 1923, 1930; Kleine, 1926). The concept of *Diatrichalus* was originally based on the presence of four longitudinal elytral costae, in contrast with nine costae in other trichaline genera (Kleine, 1926; Pic, 1930). The generic limits of this genus were redefined using genitalia, and the loss of secondary costae is assumed in several unrelated species (Bocak, 2001). The present DNA dataset contains only a single *Diatrichalus* with absent secondary costae (Fig. 1A). A similar loss of secondary costae was identified in some Afrotropical *Cautires* (Sklenarova, Kubecek & Bocak, 2014) and in an undescribed species of *Schizotrichalus*. Net-winged beetles are soft-bodied and therefore the elytral costae apparently have a strengthening function. The arrangement of the costae depends on body size and shape. The costae are commonly reduced in species with very slender or small bodies such as in Dilophotes (Lycidae: Dilophotini; Bocak & Bocakova, 2008).

### Male genitalia

The limits of most genera are currently based on the morphology of genitalia which is more reliable than external phenotypic characters. *Diatrichalus* has an exposed and complex internal sac (Figs. 5A–5C), *Lobatang* has a rod-shaped basal part of the internal sac (Figs. 5D, 5E and 5G–5I), *Eniclases* has the characteristic pigmented dorsal keel in the phallus (Fig. 5M) and *Flabellotrichalus* has the membranous, pigmented internal sac with a y-shaped basal part (Figs. 5N–5P). These characters were constant in respective genera and enable reliable identification, but they provide no information about deep relationships. Two sickle-like thorns at the base of the internal sac are present in *Trichalus* and *Microtrichalus* (Figs. 1B, 1C, 5F, 5J and 5K) and the preferred molecular phylogenetic hypothesis indicates their independent origin although with modest support (Fig. 1A). The presence of thorns in the internal sac is the principal character supporting their relationships in morphology-based analyses (Figs. 1B and 1C). Similar thorns are known in some *Synchonnus* (Kusy, Sklenarova & Bocak, in press; Kusy, 2017) and various members of distantly related genera of Metriorrhynchini, e.g., *Cautires* (Jiruskova, Motyka & Bocak, 2016).

### Female genitalia

The female genitalia provide additional information consistent with the molecular phylogenetic analyses. The strongest phenotypic character supporting the relationships among some trichaline genera are the dorsally attached lateral glands which define the clade (*Eniclases* + *Schizotrichalus*)((*Trichalus*, *Flabellotrichalus*) *Microtrichalus*). Other characters define the limits of genera, but do not contribute to the definition of more extensive clades. *Diatrichalus* has a characteristically long spermatheca (Fig. 6B) and all *Microtrichalus* have a pair of pockets in the middle part of the vagina and a slim unpaired ventral gland at the base of the vagina (Fig. 6H). With well-defined *Microtrichalus*, the genus *Trichalus* is left without any synapomorphy and its monophyly and relationships can be recovered only by molecular analyses (Fig. 1A).

## Conclusion

The phylogeny of the trichaline clade is separately recovered from morphology and molecular data, but neither analysis robustly solves all relationships. The deepest nodes in our phylogenies remain weakly supported by morphology, and only molecular analyses provide a stable topology with relatively high support for critical nodes (Figs. 1A–1C). The terminal clade of *Eniclases*, *Schizotrichalus*, *Trichalus*, *Flabellotrichalus,* and *Microtrichalus* is unambiguously supported by the unique morphology of vaginal glands, but only weakly so by the molecular data. The limits of all genera are congruently supported by morphological synapomorphies and molecular phylogenetic analyses, but their robustness differs. *Diatrichalus* is well-delimited by several morphological characters but this clade receives only a low statistical support in our molecular analyses. The least supported genus-rank node is *Trichalus* (Fig. 1A), which is morphologically defined only by the absence of some phenotypic characters when compared with *Flabellotrichalus* and *Microtrichalus*. Similarly, this node obtains low statistical support in the molecular analyses (Fig. 1A).

The phenotypic characters can be misleading when similar structures evolve repeatedly or are so simplified that we are unable to identify homologues. Unexpectedly, the anterolateral pronotal carinae, lost in other trichaline genera, re-evolved in *Eniclases* and *Schizotrichalus*. Almost all trichaline species are unpalatable and aposematically colored, and due to their memberships in mimetic rings, the unrelated species can have similar body sizes and shapes (Bocak & Yagi, 2010). These homoplasious phenotypes attest to the strength of natural selection (Bocek & Bocak, 2016) and the traditionally used morphological characters, such as pronotal carinae, elytral costae and the shape of pronotum, display high intra-generic variability which might be caused by an independent origin of similar traits due to selective pressure. Further, the molecular phylogeny suggests repeated origins of flabellate antennae, which play a role in sexual communication. To summarize, the evaluation of both molecular and morphological signals is very valuable in net-winged beetles and their congruence should be evaluated whenever possible. Future studies can refine the trichaline classification, but a large part of the trichaline diversity has already been included in current analyses and we believe that the substantial rearrangements are improbable.

## Supplemental Information

10.7717/peerj.3963/supp-1 Supplemental Information 1 Supplementary File 1. Aligned DNA dataset. The aligned molecular 3-gene dataset.

10.7717/peerj.3963/supp-2 Supplemental Information 2 Supplementary File 2. Morphological dataset. Morphological dataset used for Maximum Parsimony analysis of trichaline relationships.

10.7717/peerj.3963/supp-3 Supplemental Information 3 Supplementary Fig. 1. Bayesian tree. Molecular phylogenetic reconstruction of trichaline relationships using Bayesian inference.

10.7717/peerj.3963/supp-4 Supplemental Information 4 Supplementary Fig. 2. Maximum parsimony trees. Morphology-based phylogenetic reconstruction of trichaline relationships using Maximum Parsimony. (A–C) Three equally parsimonious tree.

## Additional Information and Declarations

### DNA Deposition

The following information was supplied regarding the deposition of DNA sequences: The sequences used here are accessible via GenBank accession numbers: MF288149–MF288557.

### Data Availability

The following information was supplied regarding data availability: The raw data has been supplied as Supplemental Dataset Files.

### New Species Registration

The following information was supplied regarding the registration of a newly described species: Publication LSID: urn:lsid:zoobank.org:pub:BCDB57BC-DF3E-42A8-AB6D-2DCAB44799F3 Maibrius: urn:lsid:zoobank.org:act:0A2E45FB-72DB-49E7-BD7C-BC792072B106 Flabellotrichalus horaki: urn:lsid:zoobank.org:act:86069ACA-BC85-4865-847B-2EB421DC3BC3
