# Supplementary figures and images for: The comparison of molecular and morphology-based phylogenies of trichaline net-winged beetles (Coleoptera: Lycidae: Metriorrhynchini) with description of a new subgenus
Source: PeerJ. 2017 Oct 23;5:e3963. doi: 10.7717/peerj.3963 (PMC5657417; doi:10.7717/peerj.3963)

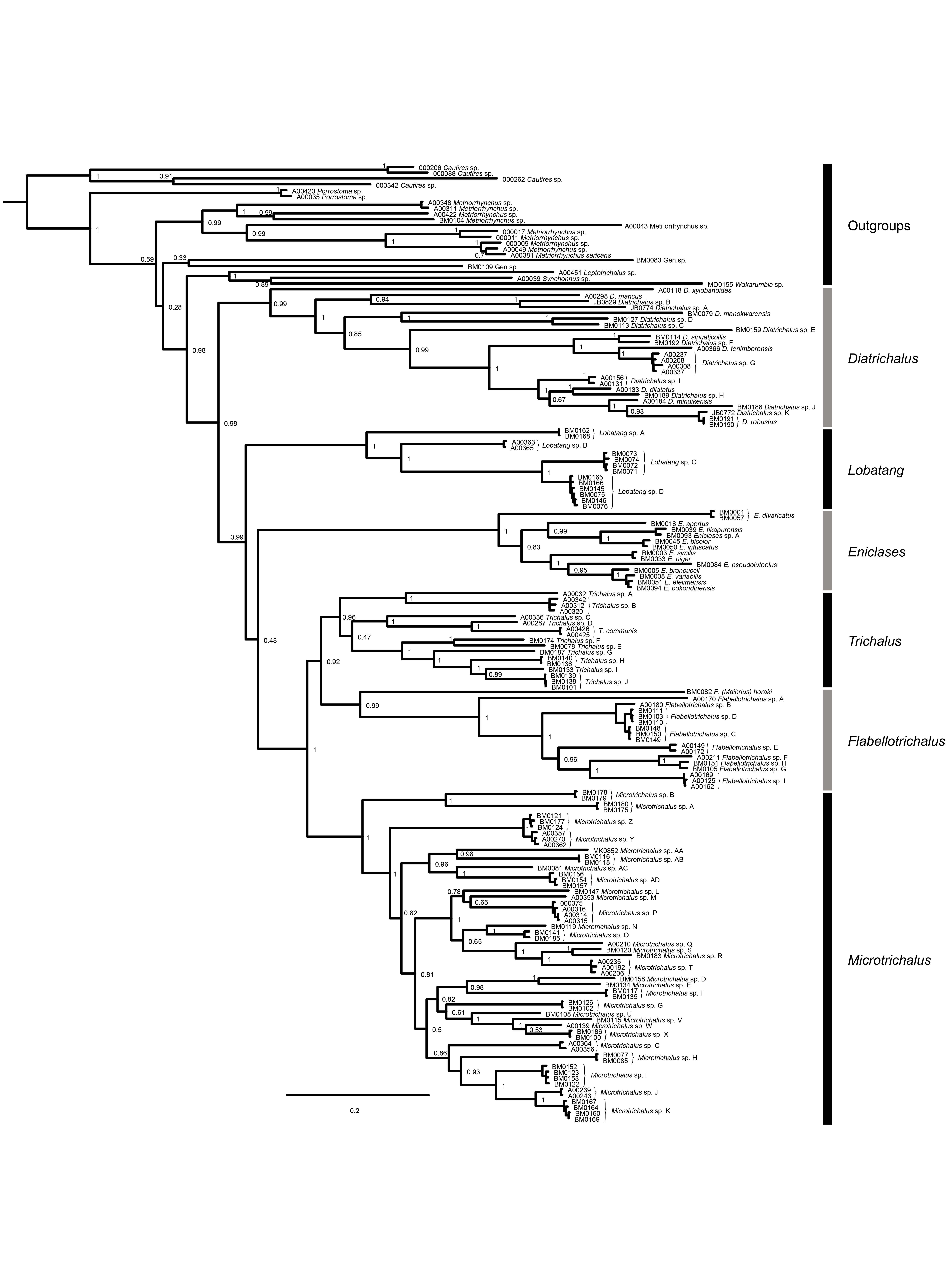

Supplement: Supplemental Information 3 — Molecular phylogenetic reconstruction of trichaline relationships using Bayesian inference. [file peerj-05-3963-s003.png]

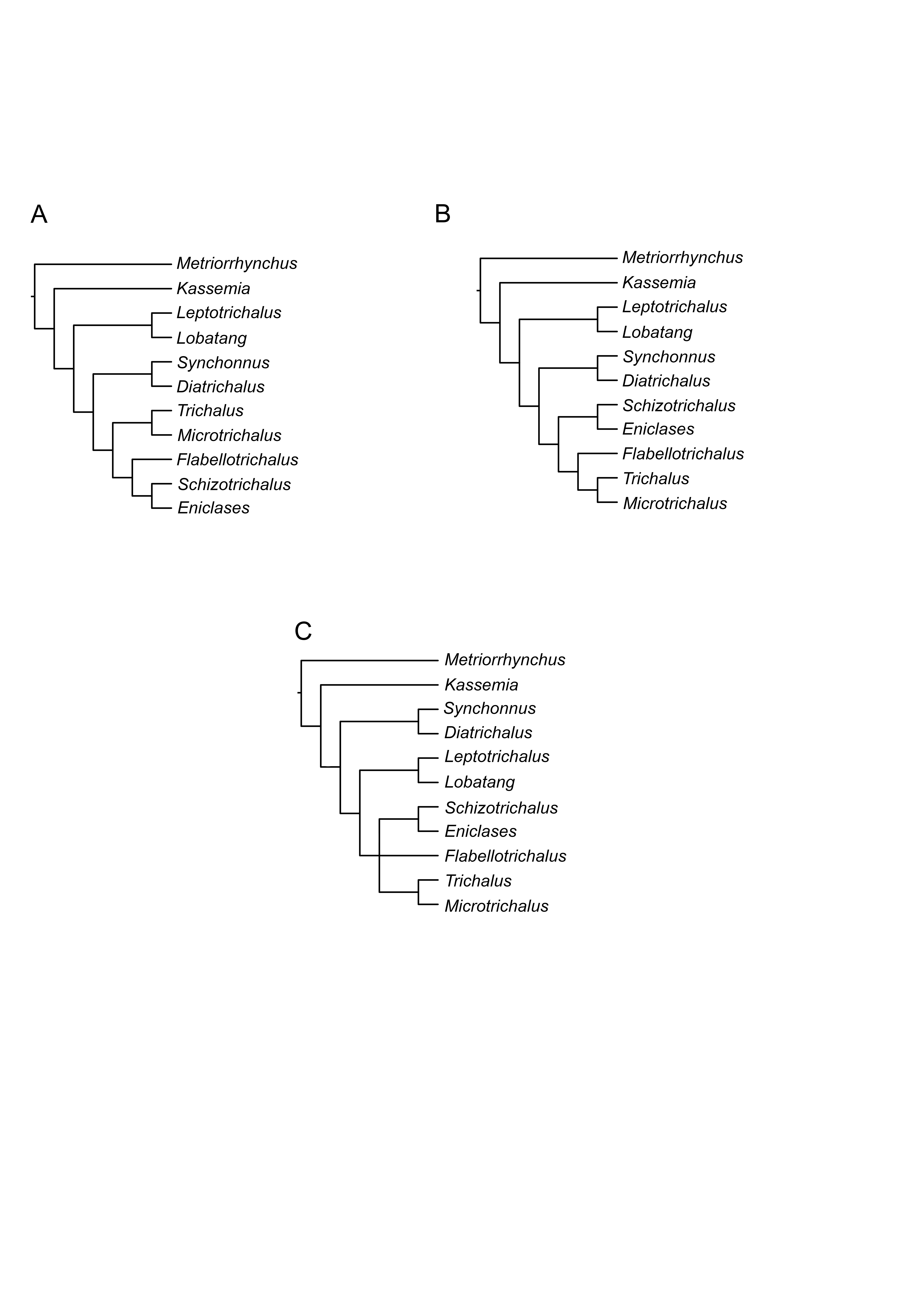

Supplement: Supplemental Information 4 — Morphology-based phylogenetic reconstruction of trichaline relationships using Maximum Parsimony. (A–C) Three equally parsimonious tree. [file peerj-05-3963-s004.png]
